# Real-Time Foot Height Estimation and Activity Classification Using a Foot-Mounted IMU Implemented on a Smartphone

**DOI:** 10.3390/s26103166

**Published:** 2026-05-16

**Authors:** Ehsan Sharafian Moghaddam, Babak Hejrati

**Affiliations:** Department of Mechanical Engineering, University of Maine, Orono, ME 04469, USA; babak.hejrati@maine.edu

**Keywords:** foot clearance, drift correction, zero height change, object crossing, locomotion activities

## Abstract

Wearable sensors are transformative tools for continuous gait assessment in daily life. Tripping, a leading cause of falls, is closely linked to inadequate foot clearance, making accurate foot height measurement critical for fall risk evaluation. Inertial measurement units offer a practical solution for foot trajectory reconstruction; however, conventional drift correction methods such as zero-velocity updates fail to adequately address cumulative height errors. Recent kinematic constraint-based approaches improve height accuracy but remain limited to offline processing and lack simultaneous activity classification. To address these gaps, we developed a real-time, single-IMU system for continuous foot height trajectory reconstruction with simultaneous classification of five locomotion activities deployed on a smartphone. Twenty healthy adults were recruited for model training and independent validation. Level walking maintained ground reference (0.0 cm, 95% CI: [−1.8, 1.8] cm), cumulative height errors remained below 1.1 cm across ramp and stair negotiation with a mean absolute error of 0.42%, and obstacle clearance was quantified. The system achieved 96.08% overall classification accuracy with less than one gait cycle latency. Toe height was estimated through rigid-body transformation with comparable accuracy to the foot height. This framework provides a practical foundation for real-time gait intervention and fall prevention applications.

## 1. Introduction

Wearable health monitoring technologies are transforming healthcare delivery for people with chronic diseases by enabling continuous monitoring of health signals and activity patterns during everyday life, replacing frequent clinic visits [1,2]. These devices offer lower cost, portability, and accessibility, enabling decentralized care and early detection of health deterioration [3,4,5]. By integrating longitudinal data with AI/ML algorithms, wearable systems support personalized health management and remote clinical oversight [6,7,8,9], and are increasingly established as practical tools across chronic disease management, rehabilitation, and care [10].

Falls represent a major public health challenge, with significant consequences including injury, hospitalization, loss of independence, and substantial healthcare costs [11,12]. While technology historically focused on fall detection after occurrence [13], a newer paradigm emphasizes fall prediction and prevention through longitudinal monitoring of gait and balance [14,15]. Instrumented gait measures such as speed, variability, and dual-task performance are consistently associated with fall risk [15,16,17,18,19]. Wearable sensors can now capture gait continuously in daily life, enabling proactive monitoring systems that combine gait features with contextual risk factors to predict and prevent falls [14,20], offering a critical path from episodic clinic-based screening to continuous real-world risk monitoring [15].

Tripping, defined as unexpected foot contact with the walking surface or an object that generates sufficient momentum to destabilize the walker, is the leading cause of falls [21,22]. Minimum foot clearance (MFC), the local minimum vertical distance between the swing foot and the ground at mid-swing, serves as a critical biomarker of tripping risk [21,23]. Lower MFC or high MFC variability increases the probability of striking obstacles, and older adults often show greater MFC variability and more very low clearances strongly linked to elevated fall risk [21,24]. MFC is often measured using optical motion capture in gait laboratories [21,22], providing high-accuracy 3D kinematics. However, this method is confined to controlled settings, expensive, and unsuitable for continuous monitoring in real-world environments, where variability in terrain, footwear, and environmental hazards is a critical risk factor for tripping in community settings [25]. To address these limitations, wearable inertial sensors combined with personalized foot geometry aim to provide accurate estimation of whole-foot MFC during level and uneven walking outside a laboratory, enabling continuous real-world monitoring of foot clearance [26,27].

Unlike optical motion capture, inertial measurement units (IMUs) require no cameras, calibrated volumes, or controlled lighting, enabling measurements in any environment [28]. IMUs are now the most widely used wearable sensor for gait analysis [29], demonstrating good-to-strong agreement with gold-standard references [30]. Because they are body-worn and self-contained, IMUs are suitable for free-living deployment, capturing gait adaptations in real-world environments [31]. Given these advantages, there is a critical need to investigate how IMU technology can be leveraged to monitor foot clearance (FC) in real-world conditions, addressing the technical challenges that have limited its use as an essential fall-risk biomarker.

Accurate FC estimation from a foot-mounted IMU requires double integrating of acceleration to obtain position; however, any sensor bias, scale error, and noise accumulated through this integration result in cumulative drift in velocity and height estimation over each stride [32,33,34,35]. For MFC, where true vertical distances are critically small, even minor uncorrected bias produces height error that can completely mask low clearances, making drift control essential. Common drift mitigation relies on zero-velocity updates (ZUPT) during stance, typically within extended Kalman filter (EKF) or error-state Kalman filter (ESKF) frameworks. Yuan et al. [32] proposed ESKF-based ZUPT using midpoint integration. In contrast, Zhang et al. [34] and Wagner et al. [36] introduced constraint methods, including planarity and height constraints, to address residual drift. Tong et al. [37] combined unscented Kalman filtering with zero-velocity detection. Multi-sensor fusion approaches by Huang et al. [38] and Liu et al. [39] further reduce drift but require additional hardware unsuitable for single-IMU deployment. However, Wang et al. [33] and Ju et al. [35] reported that heel strike impacts introduce an additional error source due to impulsive foot-ground forces, which generate acceleration spikes greater than IMU dynamic range. This creates velocity discontinuities that cannot be removed by standard ZUPT or ESKF formulations. Despite progress, current approaches are limited by computational complexity unsuitable for resources-constrained wearable devices [32,40], multi-sensor requirements [41,42], residual height drift [32,33,34], and primarily offline implementations [39,43,44].

Falls and tripping risk depend strongly on terrain, as stairs and ramps impose greater biomechanical demands than level ground and are associated with elevated fall and injury risk in older adults [33,45]. Meaningful FC assessment must therefore be activity-specific, as minimum safe clearance over a stair or ramp differs from that on smooth level ground. Context-aware systems increasingly use wearable sensors to relate gait patterns and fall risk to specific activities [45], with real-time activity detection critical for safety monitoring [46] and terrain-appropriate assistance in powered exoskeletons and prostheses [33,47]. Human activity recognition (HAR) using a single foot-mounted IMU and machine learning techniques has achieved high classification accuracy [29,33,48]. However, knowledge gaps still remain, as most HAR studies either classify terrain without reconstructing precise foot trajectories or vice versa [29,33]. High-accuracy systems often rely on multi-sensor setups that increase cost and reduce compliance [33,45,49], and many systems require offline processing unsuitable for wearable deployment [29,50]. For practical purposes, clearance monitoring systems should integrate activity classification with clearance measurement in a unified framework, using minimal sensor configurations [45,50]. This motivates single-sensor solutions that combine real-time activity classification with drift-mitigated trajectory reconstruction to provide context-aware indicators of trip risk in everyday environments.

Given the importance of foot clearance monitoring, activity classification, and tripping risk assessment for fall prevention, a practical system must operate in real-time to enable timely interventions. Reviews by Prasanth et al. [29] and Hutabarat et al. [51] highlight that many systems rely on offline post-processing with limited free-living validation. Real-time systems must satisfy stringent requirements: low-latency gait-event prediction [52], computational efficiency for constrained devices [18,53], and on-device inference [54]. Real-time capabilities are essential for rehabilitation, assistive devices, and fall-detection applications [55], and can enable auditory or haptic cues for coarse foot clearance monitoring on hazardous terrain transitions. However, mobile deployment faces limited processing power, battery constraints, and streaming reliability concerns [56]. Zafar et al. [57] emphasize that few systems simultaneously achieve high accuracy, real-time operation on constrained hardware, and free-living validation. Notably, Wang et al. [33], the most comparable prior work, demonstrated accurate foot trajectory reconstruction and terrain classification across five locomotion activities using a single foot-mounted IMU; however, their method operates entirely offline. Despite substantial progress in IMU-based gait analysis and activity recognition, to the best of our knowledge, no previous work has simultaneously demonstrated real-time, drift-corrected foot clearance measurement using a single IMU suitable for continuous, free-living monitoring that integrates activity classification with accurate trajectory reconstruction.

Given the existing limitations in correcting cumulative drift in IMU-based height estimation, reliance on offline processing, multi-sensor requirements, and the absence of real-time foot height monitoring as a practical tripping risk indicator, this study aims to develop and validate a real-time, single-IMU system for simultaneous activity classification and FC measurement on a smartphone suitable for practical clinical and home-based applications.

The contributions of this study include:

- Development of a smartphone-based Android application for real-time stride-by-stride foot clearance measurement and activity classification, integrating the zero height change (ZHC) constraint for drift correction and heel strike velocity error for activity classification, enabling on-device processing without reliance on cloud computing.
- Estimation of toe position through rigid-body transformation from IMU position and orientation.
- Demonstration of the foot height measurement accuracy through experiments, including cumulative height validation during stair and ramp negotiation.

While the motivation for this work is rooted in fall prevention for older adults, initial validation in a healthy population with well-characterized gait patterns allows rigorous algorithm development and system verification before introducing the additional complexity of age-related or pathological gait variability. The algorithmic framework validated here provides a transferable foundation for subsequent validation across broader populations, consistent with comparable prior works in this domain [26,33,48]. These contributions advance upon these works by combining drift-corrected trajectory reconstruction with adaptive, real-time activity classification in a computationally efficient, single-sensor, smartphone-based implementation suitable for clinical deployment and real-world application.

## 2. Methods

### 2.1. Kinematic Model

The kinematic model serves as the foundation for reconstructing motion trajectories from inertial sensor data. To model the kinematics of the activity, data measured by the IMU must be considered. The kinematic model is based on linear acceleration measurements from the IMU. Depending on the measurement configuration, acceleration can be captured in different reference frames, such as the global world frame or the body frame of the IMU. In this work, following a general approach, linear acceleration is expressed in the body frame of the IMU [33,58,59,60]. In the general IMU configuration, the sensor measures total acceleration in its body coordinate frame, which includes both motion-induced acceleration and gravitational acceleration. Therefore, to obtain pure kinematic acceleration, the gravitational component must be removed after transforming the measured acceleration from the body frame to the global frame of the system.

(1) ag(k)=Rbg(k)×ab(k)−gg

where *k* denotes the sample index within each stride, ag(k) is the calculated linear acceleration at sample *k* in global coordinates, ab(k) is the measured linear acceleration at sample *k* in the local body coordinate frame of the IMU, and gg is the gravity vector in global coordinates and can be considered as ${\left[0\;0\;|g|\right]}^{T}$. The term Rbg(k) represents the rotation matrix that transforms vectors from the body coordinate frame to the global coordinate frame. This rotation matrix can be constructed using either Euler angles or quaternions obtained from the IMU orientation estimation [60,61].

It is important to note that the accuracy of Rbg(k) directly affects the quality of trajectory reconstruction, as attitude errors introduce gravity leakage into the estimated kinematic acceleration components, degrading both velocity and position estimates. In this work, orientation estimates are obtained from the Xsens DOT onboard sensor fusion filter (Movella Technologies B.V., Enschede, The Netherlands), which fuses triaxial accelerometer, gyroscope, and magnetometer measurements using a Kalman-based attitude and heading reference algorithm to output quaternions at 60 Hz [62]. This filter employs gyroscope integration as the primary orientation propagation mechanism during dynamic phases of the gait cycle, particularly during mid-swing, when foot angular velocity is high, and the accelerometer cannot serve as a reliable gravity reference, while incorporating accelerometer-based corrections during quasi-static stance phases to bound long-term gyroscope drift. This design ensures convergence and reliable pitch and roll estimation under the dynamic conditions characteristic of all five locomotion activities examined in this study.

Based on the assumption that gait parameters exhibit repetitive patterns, the continuous data stream can be segmented into stride-based windows. In this study, gait segmentation is performed by identifying the zero-velocity point (ZVP), which corresponds to the flat-foot phase during walking when the foot is stationary. Each stride window begins at one ZVP and ends at the subsequent ZVP, effectively capturing one complete gait cycle. A detailed description of the stride segmentation algorithm is provided in Section 2.3. It should be noted that these formulations apply to a single IMU mounted on the dorsal surface of the foot. Further details regarding sensor placement and experimental setup are discussed in the following sections. With the linear acceleration expressed in the global frame, the linear velocity can be calculated through numerical integration of acceleration samples from the previous sample and the beginning of each stride. The velocity at sample *k* is computed as:

(2) vg(k)=vg(k−1)+ag(k)×Δt=vg(k−1)+Rbg(k)×ab(k)−gg×Δt

where Δt is the sampling time interval corresponding to the measurement frequency of the IMU, vg(k) is the linear velocity in the global coordinate frame at sample *k*, and vg(k−1) is the velocity at the previous sample.

#### 2.1.1. Linear Velocity Correction

The objective of velocity correction is to eliminate the residual offset in velocity and position that accumulates by the end of each stride. To address this issue, the sources of error must be identified. One primary source of error arises from imperfections in acceleration measurements. As noted by Wang et al. [33], these errors can stem from sensor noise, bias instability, and other systematic measurement errors that affect the recorded acceleration data. To simplify the computational approach, an acceleration bias parameter $a_{bias}$ is introduced to represent the aggregate measurement error for each stride. The acceleration bias has the same dimensions as linear acceleration (m/s^2^) and is estimated independently for each stride window. By estimating and compensating for this stride-specific acceleration bias, the proposed method aims to eliminate the velocity drift and position offsets that would otherwise accumulate at the end of each stride.

In addition to acceleration bias, the impact event at heel strike can introduce substantial errors in the kinematic model [33]. This heel strike impact represents a previously unaddressed source of velocity and position error in conventional methods such as ZUPT [63] and ESKF-based [32] reconstruction. To account for the heel strike impact, a heel strike velocity correction term is introduced, which has dimensions of velocity (m/s). Within each stride window, the heel strike event generates an impulsive force that manifests as a discontinuous velocity change in the kinematic model. This velocity discontinuity, denoted as $v_{error,hs}$, represents the instantaneous velocity error induced by the impact. The complete formulation for velocity difference must therefore include both the acceleration bias effect and the heel strike velocity error. Since all formulations are expressed in the global coordinate frame (*g*), the heel strike velocity correction vector is primarily oriented in the vertical direction. The full step-by-step derivation of the velocity difference equations is provided in Appendix A. The key result, for any sample *k* occurring after the heel strike instant ($k_{hs}$), is:

(3) Δvg(k)=vdriftg−vdriftcorrg=La(k)·abias·Δt+00verror,hs,ifk>khs

where vdriftg and vdriftcorrg are the uncorrected and corrected velocity drifts at sample *k*, respectively; $a_{bias}$ is the stride-specific acceleration bias vector; and La(k)=∑j=1kRbg(j) represents the cumulative sum of rotation matrices over the stride from the beginning to sample *k*. Beyond correcting velocity drift, the per-stride estimation of $a_{\mathrm{bias}}$ also provides a mechanism for absorbing residual attitude-induced systematic errors introduced by gravity leakage, further reducing their impact on the reconstructed trajectory.

#### 2.1.2. Height Correction

After calculating the linear velocity, the position of the IMU at each sample can be determined through numerical integration. Following the same discrete integration approach, the position vector is computed as [33,63]:

(4) pg(k)=pg(k−1)+vg(k−1)·Δt+12Rbg(k)·ab(k)−g·Δt2

where pg(k) represents the position vector of the IMU in the global frame at sample *k*.

The objective of position correction is to minimize the drift and offset that accumulate by the end of each stride. The complete position difference, including both acceleration bias and velocity heel strike effects, is derived in Appendix A. The result for any sample *k* after the heel strike moment is:

(5) Δpg(k)=pdriftg−pdriftcorrg=Lv(k)·abias·Δt2+00verror,hs·(k−khs)·Δt,for1<khs<k

where Lv(k)=∑j=1kLa(j)−12Rbg(j).

With the velocity and position difference equations (Equations (4) and (5)) established, the complete kinematic model is now ready to be formulated. In the following section, constraints based on gait assumptions will be applied to solve for the bias parameters.

#### 2.1.3. Zero Height Change (ZHC) Model

To formulate the kinematic model and solve for the bias parameters, several fundamental assumptions based on gait biomechanics must be established.

**Assumption 1: Single Heel Strike Event**. Each gait cycle contains exactly one heel strike event. The timing of this heel strike moment ($k_{hs}$) must be identified for the kinematic formulation, regardless of whether it is detected in real-time or considered as an estimation moment of it.

**Assumption 2: Zero Velocity Constraint**. The foot velocity at the beginning and end of each stride must be zero. Stride segmentation is based on identifying zero-velocity points (ZVP) that correspond to the flat-foot phase of gait, when the entire foot is in contact with the ground. While multiple instants during the stance phase may exhibit near-zero velocity, the most reliable ZVP is selected as the stride boundary.

**Assumption 3: Zero Height Change Constraint**. For walking on level ground, the vertical (z-component) position of the IMU at the beginning of the stride must equal the vertical position at the end of the stride. In other words, the height difference between the start and end of each stride on level terrain should be zero. Therefore, the proposed model is referred to as the zero height change (ZHC) model.

Moreover, obstacle crossing as a part of the experiment in this study is naturally accommodated within this framework, as the foot departs from and returns to the same ground level within a single stride, satisfying the ZHC constraint. The maximum stride height is therefore fully preserved as a measure of obstacle clearance.

Based on Assumption 2, the corrected velocity at the beginning and end of each stride must be zero. Applying this constraint to the velocity difference equation from Equation (3) at the end of a completed stride with *N* samples yields:

(6) Δvg(N)=vdriftg(N)−vdriftcorrg(N)=vdriftg(N)=La(N)·abias·Δt+00verror,hs

where $v_{drift}^{corr}\left(N\right)=0$ due to the zero-velocity constraint, and $v_{drift}\left(N\right)$ represents the uncorrected velocity drift at the end of the stride.

For position correction over a stride of *N* samples, only the vertical (z) component of the position difference equation is required. According to Assumption 3, walking on level ground implies zero height change between the start and end of the stride. To satisfy this constraint through our corrected position, the height component of the position drift must be eliminated. The height difference equation incorporating both acceleration bias and heel strike effects becomes:

(7) Δpzg(N)=pdrift,zg(N)−pdrift,zcorrg(N)=Lv,(3,:)(N)·abias·Δt2+00verror,hs·(N−khs)·Δt,for1<khs<N

where $p_{drift,z}^{corr}\left(N\right)$ represents the corrected height drift at the end of the stride, which should equal zero for level-ground walking, and $L_{v,(3,:)}$ denotes the third row of $L_{v}$.

Combining the velocity constraint (Equation (6)) and the height constraint (Equation (7)), the complete system can be expressed in matrix form:

(8) Δvg(N)Δpzg(N)4×1=La(N)·ΔtNLv,(3,:)(N)·Δt2(N−khs)·Δt4×4abiasverror,hs4×1→thenabiasverror,hs4×1=La(N)·ΔtNLv,(3,:)(N)·Δt2(N−khs)·Δt4×4−1Δvg(N)Δpzg(N)4×1

The summation matrices $L_{a}$ and $L_{v}$ can be computed recursively for computational efficiency:

(9) La(k)=La(k−1)+Rbg(k)Lv(k)=Lv(k−1)+La(k)−12Rbg(k)

The resulting system consists of four equations (three equations from the velocity constraint and one from the height constraint) and four unknowns (three components of $a_{bias}$ and one-dimension $v_{error,hs}$), yielding a fully determined linear system. With these equations established, the acceleration bias $a_{bias}$ and heel strike velocity error ($v_{error,hs}$) can be calculated for each stride by solving the system of constraint equations. The numerical stability of this matrix inversion was assessed through a condition number analysis, confirming that the matrix remains well-conditioned under realistic IMU noise conditions (Appendix F).

While both $a_{bias}$ and $v_{error,hs}$ are calculated at the end of stride and used for trajectory reconstruction, only $v_{error,hs}$ is employed for activity classification, as it captures terrain-specific heel impact characteristics through a single scalar parameter, its sign distinguishes ascent from descent, and its magnitude separates level walking from ramp and stair activities reducing classification complexity. Note that Equation (8) assumes level-ground walking (Assumption 3 valid), so it is initially applied to all strides to compute bias parameters for activity detection. Once activity type is identified, the appropriate reconstruction is selected: for level-ground walking, the full system (Equation (8)) is applied, enforcing both Assumptions 2 and 3 to minimize drift in velocity and height; for non-level activity (ramps and stairs), only the velocity constraint (Equation (6)) is applied, allowing natural height changes while maintaining zero-velocity at stride boundaries.

The ZHC constraint is applied as a universal initialization step at every stride, regardless of terrain, so the classification is not based on a prior terrain assumption. Equation (8) is solved under the level-ground assumption to compute $a_{bias}$ and $v_{error,hs}$ at each stride boundary, and $v_{error,hs}$ is free to take any value without memory of previous strides, so classification emerges from the physics of the heel strike impact. Any misclassification at a terrain transition is self-limiting: both parameters are re-estimated fresh at every ZVP, and in typical ambulation, non-level terrain is always entered from and returned to level-ground segments where the ZHC constraint re-engages naturally, resetting any residual drift.

#### 2.1.4. ZHC vs. ZUPT: Motivation for Height Constraint

**Figure 1:**
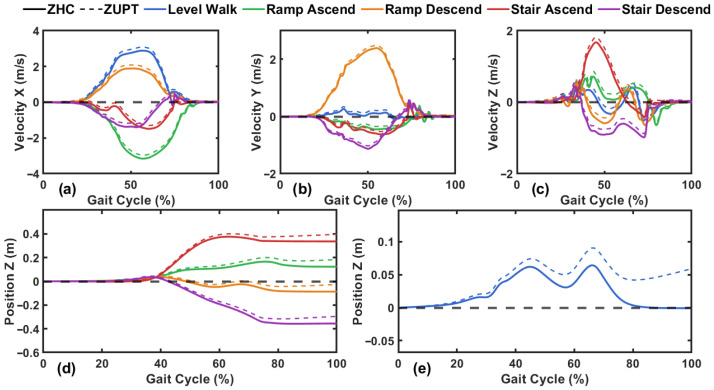
Comparison of ZHC (solid) and ZUPT (dashed) methods across five locomotion activities. (**a**–**c**) Velocity profiles in X, Y, and Z directions over the normalized gait cycle. (**d**) Vertical position trajectories for non-level ground activities. (**e**) Vertical position trajectories showing that ZUPT incorrectly resets height to zero at a stride on level ground, while ZHC removes the drift and residual height. The ZHC trajectory represents the retroactively corrected position, where $a_{\mathrm{bias}}$ and $v_{\mathrm{error},\mathrm{hs}}$ are solved at the end of the completed stride and applied to all samples within that stride.

Figure 1 illustrates the fundamental difference between ZUPT-based and the proposed ZHC method. As shown in Figure 1a–c, both methods enforce zero velocity at the beginning and end of each stride, effectively removing velocity drift at stride boundaries. However, this velocity correction alone is insufficient to eliminate height drift. As shown in Figure 1e, even with ZUPT applied, the vertical position at the end of a level-ground stride does not return to its initial value, resulting in a residual height offset. In contrast, the ZHC method addresses this limitation by additionally enforcing a zero height change constraint at stride boundaries during level-ground walking. As shown by the solid lines in Figure 1e, the ZHC method eliminates the drift and residual height offset during level walking. It should be noted that the ZHC trajectory in Figure 1e represents the retroactively corrected position, where $a_{\mathrm{bias}}$ and $v_{\mathrm{error},\mathrm{hs}}$ are solved at the end of the completed stride and applied to all samples within that stride.

It should be noted that, for non-level activities where the ZHC constraint is invalid, the proposed method still differs from standard ZUPT. While standard ZUPT resets velocity but leaves the accelerometer bias uncorrected, the proposed method re-estimates $a_{\mathrm{bias}}$ at every stride boundary using the velocity constraint (Equation (6)), re-anchoring the accelerometer calibration at each ZVP and preventing the cross-stride drift accumulation that characterizes standard ZUPT.

### 2.2. Activity Recognition

#### 2.2.1. Using Heel Strike Velocity Error for the Activity Recognition

As established in the previous section, the acceleration bias and heel strike velocity error can be calculated at the end of each stride. Building upon the approach proposed by Wang et al. [33], these bias parameters can be leveraged for activity classification. However, as a key modification in our work for real-time implementation, we rely exclusively on the heel strike velocity error parameter ($v_{error,hs}$) and demonstrate that accurate activity recognition and activity detection can be achieved using this single parameter alone.

**Figure 2:**
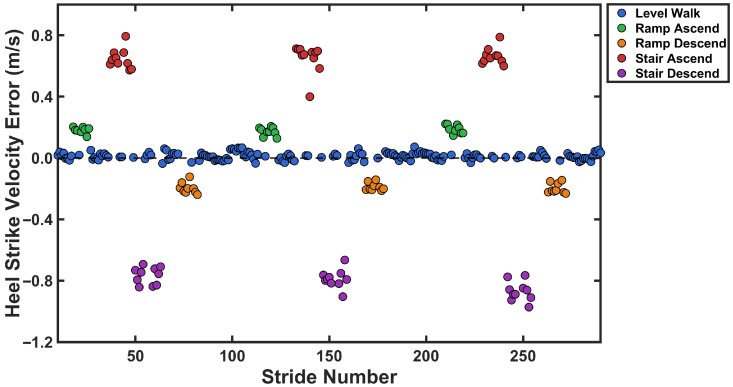
Heel strike velocity error ($v_{error,hs}$) across all strides in an experimental trial with one participant. The trial included level-ground walking, ramp ascent, ramp descent, stair ascent, and stair descent. The separation of bias values across different terrain types demonstrates that this parameter might serve as a criterion for activity recognition, with magnitude indicating terrain steepness, sign indicating direction (ascent/descent), and overall distribution distinguishing level from non-level walking.

Figure 2 shows a representative example of how $v_{error,hs}$ varies in walking on level ground, ramp ascent, ramp descent, stair ascent, and stair descent. As seen in Figure 2, three important characteristics could enable activity classification. First, the magnitude distribution of $v_{error,hs}$ could distinguish between level-ground walking and non-level terrain. Second, the sign of $v_{error,hs}$ indicated the direction of walking, separating ascending from descending activities. Third, the magnitude of $v_{error,hs}$ is differentiated between moderate inclines (ramps) and steeper inclines (stairs) in both ascending and descending directions.

For accurate activity detection using the calculated $v_{error,hs}$, shown in Figure 2, fixed threshold values could be employed [64]. However, the literature suggests that adaptive and individualized approaches are preferable to fixed thresholds, as the adaptive thresholds account for inter-subject variability and changing conditions [65]. Therefore, an adaptive, normalized, and subject-specific thresholding method was applied to use the estimated $v_{error,hs}$ for a robust and generalizable activity recognition algorithm.

#### 2.2.2. Normalized Velocity Error-Based Activity Recognition

To establish a baseline reference corresponding to level-ground walking, the interquartile range (IQR) of the heel strike velocity error magnitude is computed [66,67], defining the set of strides within the middle 50th percentile as:

(10) $$S_{IQR}=\{i:P_{25}\left(\right|v_{error,hs}\left|)\leq \right|v_{error,hs}\left(i\right)|\leq P_{75}\left(\right|v_{error,hs}\left|)\right\}$$

The baseline heel strike velocity error $\mu_{hs}$ is then computed as the median of $v_{error,hs}$ over strides within $S_{IQR}$:

(11) $$\mu_{hs}=\mathrm{median}\left(\left\{v_{error,hs}\left(i\right):i\in S_{IQR}\right\}\right)$$

This thresholding method was evaluated on the training set, yielding sufficient stride samples across all five locomotion activities to establish robust baseline parameters. Then the heel strike velocity error for each stride is normalized by subtracting the baseline reference value:

(12) $${\hat{v}}_{error,hs}\left(i\right)=v_{error,hs}\left(i\right)-\mu_{hs},\;i=1,2,\ldots ,N$$

where *N* is the number of whole strides used to compute the thresholds. This normalization serves as a calibration procedure, in which the user walks on level ground for an initial period, corresponding to the first $W_{init}=20$ strides (Section 2.3.3), allowing the system to personalize $\mu_{hs}$ to the individual’s gait characteristics and compute ${\hat{v}}_{error,hs}$. This centers the data around zero, removing the subject-specific baseline offset associated with level-ground walking, so that the normalized values ${\hat{v}}_{error,hs}\left(i\right)$ represent deviations from that individual’s typical level-ground gait pattern.

To quantify the variability of the heel strike velocity error in a robust manner, the Median Absolute Deviation (MAD) is computed:

(13) $${\mathrm{MAD}}_{hs}=\mathrm{median}(\{|{\hat{v}}_{error,hs}\left(i\right)|:i=1,\ldots ,N\})$$

The MAD is then converted to an equivalent standard deviation measure using the scaling factor for normally distributed data: $\sigma_{hs}=1.4826\times {\mathrm{MAD}}_{hs}$ where the constant $1.4826=1/\left(0.6745\right)\approx 1/\Phi^{-1}\left(3/4\right)$ is the conversion factor that makes MAD comparable to standard deviation for Gaussian distributions. Classification thresholds are established using the baseline $\mu_{hs}$ and the robust spread estimate $\sigma_{hs}$. Two threshold multipliers $k_{1}$ and $k_{2}$ (where $k_{2}>k_{1}>0$) define the boundaries between terrain types:

(14) $$\begin{array}{cc} \mathrm{For}\;\mathrm{Ascent}: & \left\{\begin{array}{c} T_{ramp}^{+}=\mu_{hs}+k_{1}\times \sigma_{hs}\\ T_{stair}^{+}=\mu_{hs}+k_{2}\times \sigma_{hs} \end{array}\right.\\ \mathrm{For}\;\mathrm{Descent}: & \left\{\begin{array}{c} T_{ramp}^{-}=\mu_{hs}-k_{1}\times \sigma_{hs}\\ T_{stair}^{-}=\mu_{hs}-k_{2}\times \sigma_{hs} \end{array}\right. \end{array}$$

Each stride *i* is classified (C(i)) based on its normalized heel strike velocity error ${\hat{v}}_{error,hs}\left(i\right)$ according to the following rules, where $T_{ramp}^{+}$ and $T_{stair}^{+}$ are the ascending activity thresholds and $T_{ramp}^{-}$ and $T_{stair}^{-}$ are the descending activity thresholds:

(15) $$C\left(i\right)=\left\{\begin{array}{cc} \mathrm{Stair}\mathrm{Ascend} & \mathrm{if}\;{\hat{v}}_{error,hs}\left(i\right)>T_{stair}^{+}\\ \mathrm{Ramp}\mathrm{Ascend} & \mathrm{if}\;T_{stair}^{+}>{\hat{v}}_{error,hs}\left(i\right)>T_{ramp}^{+}\\ \mathrm{Level}\mathrm{Ground} & \mathrm{if}\;T_{ramp}^{+}>{\hat{v}}_{error,hs}\left(i\right)>T_{ramp}^{-}\\ \mathrm{Ramp}\mathrm{Descend} & \mathrm{if}\;T_{ramp}^{-}>{\hat{v}}_{error,hs}\left(i\right)>T_{stair}^{-}\\ \mathrm{Stair}\mathrm{Descend} & \mathrm{if}\;T_{stair}^{-}>{\hat{v}}_{error,hs}\left(i\right) \end{array}\right.$$

It should be noted that the optimal values of threshold multipliers $k_{1}$ and $k_{2}$ must be determined before deployment. These parameters are optimized using supervised learning with labeled ground truth data from participants performing walking trials across various activity types. The mathematical formulation and grid search procedure for this optimization are detailed in Appendix B.

### 2.3. Real-Time System Implementation

#### 2.3.1. Zero Velocity Points (ZVP) Detection

As established in the methodology for calculating corrected position and bias parameters, which are fundamental for activity recognition and trajectory reconstruction, the zero-velocity point (ZVP) serves as the criterion for gait segmentation. This moment corresponds to the flat-foot phase of the gait cycle [68], during which the entire foot is in contact with the ground and hypothetically exhibits minimal acceleration and angular velocity. While ZVP detection is relatively straightforward in offline post-processing, identifying this moment in real time with minimal latency presents a significant challenge that requires a specialized detection strategy. Several techniques have been proposed in the literature for flat-foot phase detection, including threshold-based methods [69], machine learning approaches [70], and template matching algorithms [71]. The ZVP defines stride boundaries, with bias and error calculation and activity classification executed at each ZVP. Although Algorithm 1 supports both offline and real-time use, it is designed primarily for continuous, low-latency deployment. Detection exploits the flat-foot phase’s near-zero movement, which persists over a brief period rather than a single instant, using a sliding window to accumulate candidate samples in real-time.
**Algorithm 1** Zero Velocity Point Detection**Require:** Linear acceleration a(k), angular velocity !(k), sample time Δt, current sample index *k***Ensure:** Index of zero velocity point (ZVP) for stride segmentation  1:**Parameters:**  2:$T_{a}$: Acceleration magnitude threshold  3:$T_{\omega }$: Angular velocity magnitude threshold  4:$W_{cap}$: Target window capacity (number of samples)  5:$T_{max}$: Maximum duration since first sample in window  6:*g*: Gravitational acceleration magnitude  7:*** ***  8:**Initialize:**  9:Detection window W←∅ (empty)10:First sample time in window: $t_{first}\leftarrow$ null11:*** ***12:**for** each sample *k* **do**13:    *// Check if sample meets low-movement criteria*14:    **if** $\left|∥a(k)∥-g\right|<T_{a}$ **and** $∥\omega \left(k\right)∥<T_{\omega }$ **then**15:        **if** *W* is empty **then**16:           $t_{first}\leftarrow$ current time *// Record start of stance phase*17:        **end if**18:        Append *k* to detection window: W←W∪{k} *// Add candidate sample*19:    **end if**20:    *// Process window if full or timeout reached*21:    **if** $\left|W\right|\geq W_{cap}$ **or** (W≠∅ **and** $\left(t_{current}-t_{first}\right)>T_{max}$) **then**22:        $\min_{\omega }\leftarrow \min\left\{∥\omega \left(i\right)∥:i\in W\right\}$ *// Find minimum angular velocity*23:        $k_{zvp}\leftarrow \arg\min_{i\in W}∥\omega \left(i\right)∥$* // Select ZVP as sample with min ω*24:        Clear window: W←∅ *// Reset for next stride*25:        Reset: $t_{first}\leftarrow$ null26:        **Output** $k_{zvp}$ as detected ZVP27:    **end if**28:**end for**

The detection logic in Algorithm 1 works as follows. When both the gravity-removed acceleration magnitude ($\left|∥a(k)∥-g\right|$) and angular velocity magnitude fall below predetermined thresholds ($T_{a}$ and $T_{\omega }$, respectively), the current sample index is added to the detection window. The window continuously accumulates qualified samples until either: (1) a sufficient number of samples ($W_{cap}$) are collected, indicating a valid stance phase, or (2) the low-movement condition is violated, suggesting the end of the flat-foot period.

When the window reaches its minimum capacity, the algorithm selects the ZVP by identifying the sample with the minimum value among the accumulated candidates. Two criteria are evaluated: angular velocity magnitude and gravity-removed acceleration. Angular velocity is prioritized as the primary selection criterion due to its higher sensitivity to subtle movements during stance. Specifically, if the minimum angular velocity within the window is smaller than the minimum acceleration deviation, the ZVP is identified as the sample with the minimum angular velocity. If the detection window fails to accumulate the minimum required number of samples before the thresholds are exceeded, which may occur during rapid transitions or irregular gait patterns, the window is completed, and the detection process restarts. The threshold values and minimum window length are tuned based on typical gait event timings [48]. With this algorithm, the ZVP sample index is determined in real-time, enabling the continuous execution of bias parameter calculation and activity classification without requiring complete trial data. The detailed algorithm is presented in Algorithm 1.

The algorithm relies on four tunable parameters: $T_{a}$ (acceleration threshold), $T_{\omega }$ (angular velocity threshold), $W_{cap}$ (window capacity), and $T_{max}$ (maximum window duration). These parameters are primarily determined through empirical analysis during real-time testing of the system. The maximum duration parameter $T_{max}$ is based on established gait event timing thresholds [48], which provide physiological bounds on the duration of the flat-foot phase. The remaining parameters $T_{a}$, $T_{\omega }$, and $W_{cap}$ identify candidate samples for ZVP selection. Since the algorithm selects the minimum angular velocity sample from the accumulated candidates, the exact candidate count is non-critical; the same ZVP is selected across moderate parameter variations, provided the true minimum is captured within the window. Final parameter values were determined empirically to ensure reliable real-time performance across subjects and conditions. A sensitivity analysis of these parameters is provided in Appendix E.

#### 2.3.2. Heel Strike Detection

Beyond capturing the ZVP for real-time stride segmentation, accurate identification of the heel strike moment is essential for the proposed methodology. As established in the kinematic formulation, the heel strike sample index $k_{hs}$ represents the instant when the impulsive velocity error is applied to the system. This timing is critical for both estimating the heel strike velocity error $v_{error,hs}$ and subsequently performing activity classification. Numerous techniques have been proposed in the literature for detecting gait events such as heel strike and toe-off, typically requiring multiple sensor-specific thresholds and complex decision rules [72]. However, the stride segmentation approach adopted in this study enables a simplified detection strategy. Since each stride is bounded by two consecutive ZVPs, all kinematic data within this window corresponds to a single gait cycle. Leveraging this temporal constraint, the heel strike event can be identified as the sample exhibiting the maximum foot pitch angle within the stride window (i∈[⌊N/2⌋,N]), where the search is constrained to the second half to avoid spurious local maxima during the early swing phase.

This approach is based on the biomechanical observation that the foot reaches its maximum forward inclination at or near the instant of heel contact with the ground. The foot pitch angle, computed from the IMU orientation data, provides a reliable indicator of this critical gait event. While certain activities, particularly stair ascent and descent, may introduce greater variability in foot kinematics [48], identifying the maximum pitch angle within each stride window consistently provides a meaningful and computationally efficient estimate of the heel strike timing required for the kinematic formulation. A validation of this detection method, including timing consistency analysis across all five locomotion activities and a sensitivity analysis assessing the robustness of the kinematic model to realistic timing uncertainty, is provided in Appendix C.

#### 2.3.3. Real-Time Foot Trajectory Reconstruction

Following the determination of ZVP and heel strike index $k_{hs}$, all components for real-time activity detection and trajectory reconstruction are established. Algorithm 2 presents the implementation, integrating stride segmentation, bias and error estimation, activity classification, and trajectory correction into a unified framework. The reconstruction operates on a stride-by-stride basis, where each stride begins at one ZVP and ends at the next. For each stride, the measured acceleration is transformed to the global frame using rotation matrices derived from the IMU orientation. The heel strike moment is identified as the sample with the maximum foot pitch angle. The drift velocity $v_{drift}\left(N\right)$ and height $p_{z,drift}\left(N\right)$ are computed by integrating the uncorrected acceleration, and the constraint equations (Equation (8)) are solved to determine the acceleration bias $a_{bias}$ and heel strike velocity error $v_{error,hs}$ to modify them.
**Algorithm 2** Real-Time Trajectory Reconstruction with Adaptive Activity Classification**Require:** Linear acceleration a(k), orientation θ(k), ZVP indices, Δt**Ensure:** Corrected trajectory $\left\{p^{corr}\left(i\right),v^{corr}\left(i\right)\right\}$ and activity class *C* for each stride  1:**Parameters:** $W_{init}=20$ (baseline strides), $k_{1}^{*},k_{2}^{*}$ (threshold multipliers)  2:**Initialize:** $n_{stride}\leftarrow 0$, $\mu_{hs}\leftarrow$ null, $\sigma_{hs}\leftarrow$ null  3:*** ***  4:**for** each stride from ${\mathrm{ZVP}}_{start}$ to ${\mathrm{ZVP}}_{end}$ **do**  5:    $n_{stride}\leftarrow n_{stride}+1$  6:***    ***  7:    Transform acceleration: ag(i)←Rbg(i)×ab(i)−gg for all *i* in stride  8:    Detect heel strike: $k_{hs}\leftarrow \arg\max_{i\in [\lfloor N/2\rfloor ,N]}\theta_{pitch}\left(i\right)$  9:    Compute drift: $v_{drift}\left(N\right)$, $p_{z,drift}\left(N\right)$ using Equations (2) and (4)10:    Solve for bias: $a_{bias}$, $v_{error,hs}$ from Equation (8)11:***    ***12:    **if** $n_{stride}\leq W_{init}$ **then**                                                                 ▷ Baseline estimation phase13:        Store $v_{error,hs}\left(n_{stride}\right)$14:        **if** $n_{stride}=W_{init}$ **then**15:           Compute $\mu_{hs}$, $\sigma_{hs}$ and thresholds $T_{ramp}^{\pm }$, $T_{stair}^{\pm }$16:        **end if**17:        C← LEVEL18:    **else**                                                                                                ▷ Classification phase19:        ${\bar{b}}_{hs}\leftarrow v_{error,hs}-\mu_{hs}$20:        Classify activity: C← Apply rule from Equation (15)21:    **end if**22:***    ***23:    **if** C=LEVEL **then**24:        Use $a_{bias}$ and $v_{error,hs}$ for correction25:    **else**26:        Set $v_{error,hs}\leftarrow 0$, use only $a_{bias}$27:    **end if**28:***    ***29:    **for** each sample *i* in stride **do**                                                                       ▷ Reconstruct trajectory30:        acorrg(i)←Rbg(i)×(ab(i)−abias)−gg31:        vcorrg(i)←vcorrg(i−1)+a′g(i)·Δt32:        **if** $i=k_{hs}$ and C=LEVEL **then**33:           vzcorrg(i)←vzcorrg(i)+[00verror,hs]T34:        **end if**35:        pcorrg(i)←pcorrg(i−1)+vcorrg(i−1)·Δt+12acorrg(i)·Δt236:    **end for**37:    **Output** $\left\{p^{corr}\left(i\right),v^{corr}\left(i\right)\right\}$ and *C*38:**end for**

The algorithm employs an initial calibration period, $W_{init}$, using the first 20 strides during level ground walking to establish baseline parameters $\mu_{hs}$ and $\sigma_{hs}$. A sensitivity analysis evaluating the effect of inaccurate or unavailable calibration on classification performance is provided in Appendix D. After calibration, activity classification thresholds are computed using optimized multipliers $k_{1}^{*}$ and $k_{2}^{*}$. Each subsequent stride is classified by comparing its normalized heel strike velocity error ${\bar{b}}_{hs}$ against these thresholds. Based on the classified activity, the appropriate reconstruction model is applied. For level ground, both bias parameters are used to ensure zero height change. For ramps and stairs, $v_{error,hs}$ is set to zero ($v_{error,hs}=0$) and only acceleration bias error correction is applied, allowing natural height changes. This is due to the fact that for non-level walking activity, Assumption 3 cannot be used. The corrected acceleration is integrated to compute velocity and position, yielding accurate trajectory reconstruction throughout each stride.

It should be noted that the ZHC constraint solves for $a_{\mathrm{bias}}$ and $v_{\mathrm{error},\mathrm{hs}}$ at the end of each completed stride, meaning the corrected trajectory for stride *i* becomes available only after ZVP detection at the end of that stride, not during mid-swing. The system therefore operates on an inter-stride basis, where kinematic features from stride *i* inform feedback or adaptive responses during the subsequent stride i+1, consistent with established haptic feedback paradigms for gait training [18,73].

#### 2.3.4. Smartphone Application Deployment

To enable real-time data collection and processing, a custom Android application was developed using the Movella (Xsens Technologies B.V., Enschede, The Netherlands) IMU software development kit (SDK) version 2023. The initial versions of the Android application were developed in [73,74]. The system utilizes Xsens DOT wearable IMU sensors sampling at 60 Hz, with a Google Pixel 6a smartphone (Google LLC, Mountain View, CA, USA) serving as the development and deployment platform. The implementation workflow consisted of three phases: data collection, offline model development in MATLAB (MathWorks Inc., Natick, MA, USA), and real-time deployment on the Android platform.

**Figure 3:**
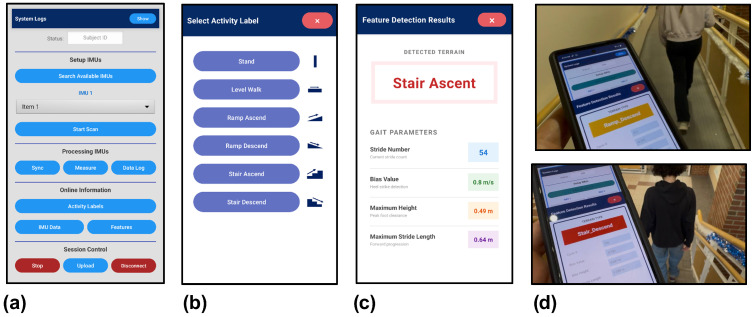
Overview of the custom Android application for real-time gait analysis. (**a**) Main interface with system controls and IMU connection management. (**b**) Activity labeling interface for ground truth annotation during training trials. (**c**) Real-time activity classification and gait feature display showing detected activity, stride number, heel strike velocity error, maximum stride height, and maximum stride length. (**d**) Application deployment during stair ascent and descent with two participants.

The developed Android application supports two operational modes, as illustrated in Figure 3. The first mode (Figure 3b) is designed for supervised data collection and ground truth labeling with the buttons corresponding to each activity. During training trials for data collection, an operator uses the application interface to manually label activity types as participants walk through structured environments. This labeled dataset is used to optimize the classification threshold multipliers as described in Appendix B.

The second mode (Figure 3c) implements real-time activity recognition and gait feature extraction. In this deployment mode, the application executes the complete processing pipeline, including ZVP detection (Algorithm 1), trajectory reconstruction with adaptive activity classification (Algorithm 2), and stride-based kinematic feature extraction, directly on the smartphone. Real-time output includes classified activity type, stride length, stride height, maximum stride height, and estimated toe clearance for each completed stride.

The system development followed a two-stage process. Initially, raw IMU data (linear acceleration, angular velocity, and orientation) were collected via the Android application and streamed to the smartphone’s internal storage. These data were subsequently processed offline in MATLAB to develop and validate the kinematic models, optimize algorithm parameters, and tune classification thresholds using the labeled training dataset. Once validated, the complete algorithm was translated from MATLAB to Java and integrated into the Android application using Android Studio. This enabled standalone real-time execution on the smartphone without requiring external computational resources. The real-time implementation maintains computational efficiency suitable for continuous operation on mobile hardware while providing stride-by-stride feedback with latency of less than one gait cycle.

Regarding the frequency at 60 Hz, it is worth mentioning that the sampling interval is approximately 16.7 ms, providing roughly 24–30 samples during a typical swing phase of 400–500 ms [29]. The minimum detectable vertical displacement per sample at this sample rate can be approximated as $\Delta p=\frac{1}{2}\times a\times \Delta t^{2}=\frac{1}{2}\times 9.81\times {\left(0.0167\right)}^{2}\approx 1.37$ mm (Based on Equation (8)), this millimeter-level resolution can be sufficient for the primary applications of our system, obstacle crossing and stair negotiation training where MFC is on the order of 10 cm or more, while acknowledging that it may limit precise localization of sub-centimeter level-ground MFC events. This sampling rate can represent a deliberate trade-off between temporal resolution and real-time computational feasibility, as higher rates would increase Bluetooth transmission load and processing burden on the smartphone, potentially compromising the real-time operation.

No additional signal conditioning was applied beyond the onboard processing pipeline of the Xsens DOT sensor, which internally samples and calibrates raw MEMS data at 800 Hz, including a low-pass filtering stage prior to Strap-Down Integration, before delivering calibrated acceleration and orientation at 60 Hz [62]. Additional post-sensor filtering was deliberately omitted for two reasons. First, causal low-pass filtering introduces a phase delay that would shift the detected heel strike timing $k_{hs}$, degrading the constraint system; zero-phase alternatives are incompatible with real-time stride-by-stride operation. Second, the heel strike impact is not noise to be suppressed but a physically meaningful event whose integrated effect is explicitly estimated as $v_{error,hs}$ at each stride boundary; aggressive filtering would attenuate this parameter, compromising both classification and trajectory correction. Any residual noise is bounded within each stride and partially absorbed by the per-stride $a_{bias}$ estimation.

### 2.4. Foot Trajectory Parameters

**Figure 4:**
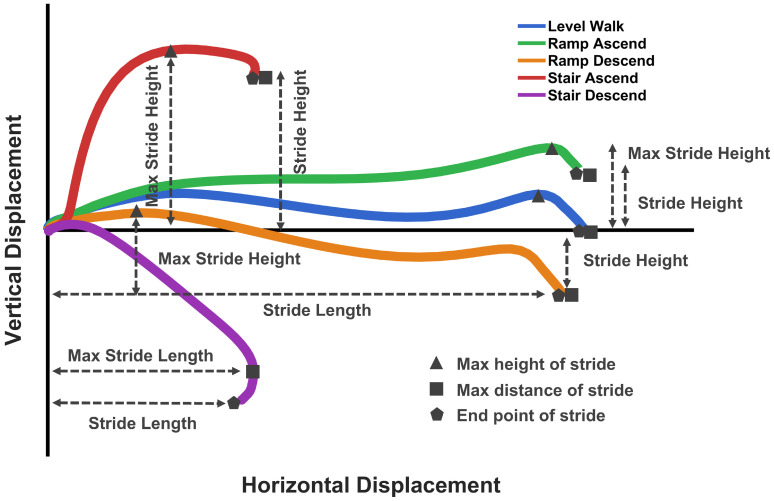
Representative stride trajectories in the sagittal plane for the activity types. Four kinematic features are annotated: stride height (vertical displacement from start to end), maximum stride height (peak vertical excursion), stride length (horizontal displacement), and maximum stride length (peak horizontal distance).

The reconstruction of foot trajectory enabled real-time computation of key kinematic features for evaluating foot placement and reducing fall risk across different activities. Four key features are defined based on the reconstructed trajectory, as shown in Figure 4.

- **Stride height** is defined as the vertical displacement between the initial and final positions of the stride:
  (16) $$\Delta h=p_{z}\left(N\right)-p_{z}\left(0\right)$$
  This metric captures the net height change during the stride and is zero for level-ground walking, positive for ascending activity, and negative for descending activity.
- **Maximum stride height** represents the maximum vertical excursion of the foot during the stride, computed as the difference between the highest and lowest points in the sample *k* of the stride:
  (17) $$h_{max}=\max_{k}\left(p_{z}\left(k\right)\right)-\min_{k}\left(p_{z}\left(k\right)\right)$$
  This feature provides a measure of foot clearance and is relevant even for level-ground walking, where stride height is zero but the foot undergoes vertical movement during swing phase.
- **Stride length** is the horizontal displacement between the initial and final foot positions:
  (18) $$L=\sqrt{{\left(p_{x}\left(N\right)-p_{x}\left(0\right)\right)}^{2}+{\left(p_{y}\left(N\right)-p_{y}\left(0\right)\right)}^{2}}$$
  This represents the forward progression achieved during the stride.
- **Maximum stride length** is defined as the maximum horizontal distance reached at any point during the stride:
  (19) $$L_{max}=\max_{k}\left(\sqrt{{\left(p_{x}\left(k\right)-p_{x}\left(0\right)\right)}^{2}+{\left(p_{y}\left(k\right)-p_{y}\left(0\right)\right)}^{2}}\right)$$
  For most activities, including level walking, ramp ascent, ramp descent, and stair ascent, the maximum stride length equals the stride length, as the foot progresses monotonically forward.

For level walking, ramps, and stair ascent, maximum stride length equals stride length because the foot continuously progresses forward. In stair descent, however, the curved downward trajectory causes the foot to reach its maximum forward position mid-stride before retracting slightly to land on the lower step, making maximum stride length greater than stride length. This is the case that the different definition of max stride length becomes meaningful.

Representative sagittal trajectories (Figure 4) illustrate the distinct biomechanical demands of each activity: minimal excursion for level walking, gradual height changes for ramps, and pronounced curved paths for stairs. These features provide quantitative metrics for assessing foot clearance and progression, overcoming the height drift limitations inherent in conventional ZUPT or ESKF methods [63]. The proposed bias/error-correction approach enables reliable real-world measurement of these clinically significant parameters.

### 2.5. Toe Clearance Estimation

The proposed trajectory reconstruction method enables real-time estimation of foot clearance during locomotion, a critical metric for assessing fall risk and gait safety. While the kinematic features such as maximum stride height provide valuable information about foot motion, all calculations are initially referenced to the IMU location on the dorsal surface of the foot. However, during level-ground walking and stair negotiation, ground contact or obstacle collision occurrence is more likely to happen at the toe, not at the IMU location. Therefore, the estimation of the toe position throughout gait cycle was investigated. To determine toe position from the known IMU trajectory, we assumed the foot as a rigid body. Although the foot exhibits some flexibility, particularly at the metatarsophalangeal joints during toe-off, this simplification provides a reasonable approximation for clearance estimation during the swing phase when collision risk is highest. Given the position and orientation of the IMU, the toe position can be calculated using rigid-body kinematics. However, an analysis of how metatarsophalangeal joint flexibility can affect toe height estimation, evaluated across locomotion activities and physiologically plausible flexion angles, is provided in Appendix G.

**Figure 5:**
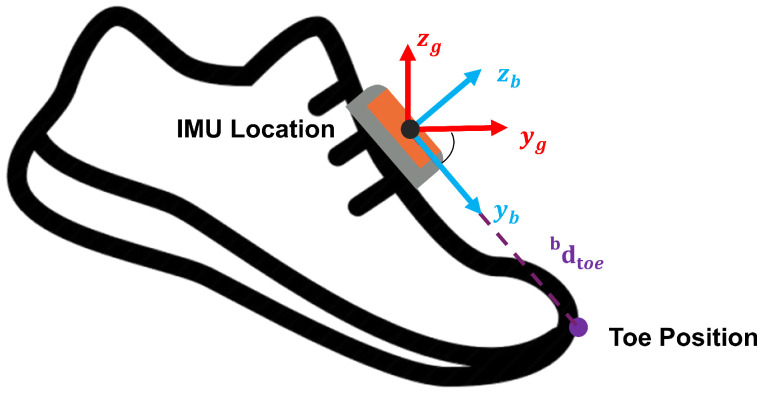
Schematic illustration of IMU and toe position relationship on the foot. The IMU is mounted on the dorsal anterior surface of the foot with its body frame axes and corresponding global frame axes. The displacement vector dtoeb connects the IMU location to the toe position in the body frame.

As illustrated in Figure 5, the IMU is mounted on the dorsal anterior surface of the foot. The body frame of the IMU defines the local coordinate system for linear acceleration measurements, which are transformed to the global frame using the rotation matrix Rbg as described in previous sections. The toe position in the global frame is calculated as:

(20) ptoeg=pIMUg+Rbg·dbwhere db=[0d0]T

where ptoeg is the toe position in the global frame, pIMUg is the IMU position in the global frame (computed from the trajectory reconstruction algorithm), and db is the displacement vector from the IMU to the toe expressed in the body frame of the foot. The displacement vector db is measured once for each subject by determining the distance from the IMU mounting location to the tip of the toe along the foot’s longitudinal axis. This measurement remains constant throughout the trial under the rigid-body assumption.

It is important to note that the proposed method provides height reconstruction (*z*-component) through the zero-height-change constraint on level ground and the bias error correction formulation. The individual horizontal position components (*x* and *y*) are subject to heading drift due to the absence of heading correction. However, the horizontal displacement magnitude (calculated as $\sqrt{x^{2}+y^{2}}$) is usable within each stride because the zero-velocity constraint at each ZVP effectively resets the accumulated drift at stride boundaries. Consequently, the stride length *L* calculated from *x* and *y* displacements within each stride window is unaffected by absolute global heading drift, as each stride begins from a fresh zero-velocity reference with no accumulated heading error from previous strides. This enables measurement of stride length and horizontal foot progression between consecutive ZVPs. This approach enables continuous monitoring of minimum toe clearance, which is a predictor of tripping risk [22,75].

Moreover, regarding the heading drift, it is important to note that it corresponds to yaw rotation about the vertical axis, which mixes only the horizontal components of the coordinate frame and does not project into the vertical acceleration component; therefore, heading drift does not directly affect height estimation. The attitude errors relevant to vertical accuracy are pitch and roll, which are bounded by the onboard accelerometer-based correction during quasi-static stance phases regardless of magnetometer availability. Furthermore, any residual attitude-induced systematic error in the vertical channel is absorbed by the per-stride $a_{bias}$ estimation at each stride boundary, preventing cumulative growth across strides.

## 3. Experimental Design and Analysis

### 3.1. Participants

Twenty healthy adults were recruited and assigned to two groups for model development and validation. The first group, the training group, consisted of 10 participants (5 females, age: 19.6±1.3 years) whose data were used to optimize classification threshold parameters and tune algorithm settings. The second group, the testing group, comprised 10 participants (5 females; age: 20.4±3.7 years) for independent validation of the real-time model’s accuracy for activity detection and foot height calculation. Upon arrival, participants were provided with a detailed description of the experimental protocol and written informed consent. The study was approved by the University of Maine Institutional Review Board (IRB protocol number 2019-0-15). The experimental tasks included walking on level ground, ascending and descending a staircase connecting two floors of a building, and walking along an inclined ramp, each repeated for a predetermined number of trials. Participants were informed they could rest at any time during the experiment and were permitted to use handrails during stair climbing or ramp walking if needed for safety or comfort.

### 3.2. Experimental Environment

The experimental protocol was conducted in a building selected to simulate typical home and community environments in daily living. This setting provided the necessary activity variety, including level walking, a staircase, and a ramp, to validate the model’s robustness in detecting all five locomotion modes. Both training and testing phases were conducted at this location. The testing environment consisted of a closed-loop pathway incorporating multiple activity types. Participants began with level-ground walking and then transitioned to a ramp segment approximately 15 m in length with a 4° inclination angle. Following the ramp, participants ascended a staircase consisting of 22 steps arranged as two flights of 11 steps with an intermediate turning platform. Each step measured 0.34 m (34 cm) in tread depth and 0.17 m (17 cm) in rise height. After reaching the upper level, participants immediately reversed direction, descended the staircase, and returned to the starting point via level-ground walking. A seating area was available for participants to rest between trials. This structured pathway was repeated multiple times, enabling comprehensive training data collection and rigorous model validation.

The building also contained a separate validation staircase consisting of 14 steps with a rise of 0.137 m per step and 0.31 m in depth, connecting two floors with a total architectural height difference of 1.921 m (14×0.137m≈1.921m). This staircase was used exclusively for the cumulative height validation experiment described in Section 3.4.1, as its total floor-to-floor height was independently measured and served as an absolute ground truth for cumulative drift assessment. To distinguish between the two staircases, the 22-step staircase is referred to as the main staircase throughout the manuscript, and the 14-step staircase is referred to as the validation staircase.

### 3.3. Data Collection and Model Training

Ten participants (training group) completed seven cycles of the closed-loop pathway, providing multiple repetitions. During these trials, ground truth activity labels were collected using the custom Android application’s labeling interface (Figure 3b). An operator followed at a safe distance, annotating activity transitions in real time by pressing the corresponding activity button at each transition onset and releasing it upon the next transition. This created timestamp markers synchronized with the 60 Hz IMU data stream, yielding a labeled dataset with precise activity boundaries.

All labeled data were saved as CSV files containing raw IMU measurements and corresponding activity annotations. These files were subsequently processed offline in MATLAB to optimize classification threshold parameters. Following parameter optimization, the complete algorithm was translated from MATLAB to Java and deployed on the Android application for real-time testing.

Ground truth activity labels were collected using the Android application’s labeling interface (Figure 3b), where an operator annotated activity transitions in real-time by pressing the corresponding activity button, inserting timestamp markers into the IMU data stream. The resulting labeled dataset was saved as CSV files and processed offline in MATLAB to optimize classification threshold parameters, after which the complete algorithm was translated to Java and deployed on the Android application for real-time testing.

### 3.4. Real-Time Testing

The testing group performed seven cycles of the experimental pathway, while the real-time Android application autonomously classified activity and calculated foot height using optimized parameters. While the model operated without manual input, an operator recorded ground-truth labels for accuracy assessment. Recognition was initiated following a 20-stride calibration period on level ground, which established the baseline normalization parameters for heel strike velocity error. Following completion of the walking trials, participants performed obstacle clearance experiments over three boxes of different heights.

To evaluate classification performance, the application’s real-time activity predictions were compared with operator-annotated ground-truth labels for each stride. A prediction was counted as correct when the classified activity matched the true label; otherwise, it was counted as incorrect. The system exhibited a classification latency of approximately 110 ms following the detection of a ZVP as the end of a stride, attributable to two sources: the inherent transmission delay between the IMU and the smartphone application, and the computational time for bias calculation and activity classification. Classification accuracy metrics, including overall accuracy and per-activity confusion matrices, were compiled after each participant completed the experimental protocol and aggregated across all testing participants for analysis.

To assess system accuracy under clinically relevant conditions, two validation experiments were designed, focusing particularly on foot height estimation. While previous offline methods have demonstrated promising trajectory reconstruction results [26,33], real-time performance must be validated to ensure practical utility for applications such as obstacle avoidance.

#### 3.4.1. Cumulative Height Accuracy During Stair and Ramp Negotiation

**Figure 6:**
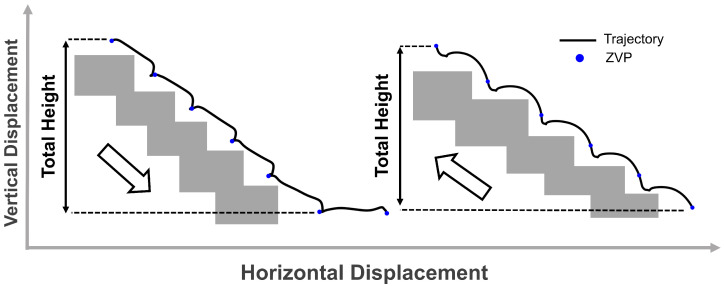
Validation of cumulative height measurement during stair descent. The reconstructed foot trajectory (solid black line) is segmented by zero-velocity points (ZVPs, blue dots) corresponding to each foot-flat phase. The total vertical displacement calculated by summing individual stride heights is compared against the known architectural height difference between floors (indicated by gray stair structure and arrow showing descent direction).

The first experiment evaluates cumulative height measurement during stair ascent/descent between building floors. As illustrated in Figure 6, participants traversed a complete staircase connecting two floors while the application continuously tracked their foot height through multiple strides. Zero-velocity points (ZVPs, marked as blue dots) segmented individual strides, and the total vertical displacement was computed by summing stride heights from the initial floor to the final floor. The calculated total height change was compared against the known height difference between floors, validating the system’s ability to integrate height changes over extended multi-stride sequences without excessive drift accumulation. The same procedure was applied for ramp ascent and descent, comparing the calculated total height difference against the known elevation change in the ramp.

#### 3.4.2. Foot Clearance Measurement During Obstacle Negotiation

**Figure 7:**
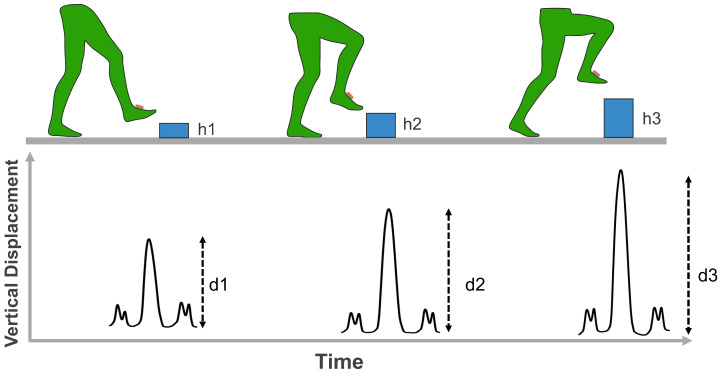
Obstacle clearance validation setup and measurement. (**Top**) Participants step over boxes of varying heights ($h_{1},h_{2},h_{3}$) during level-ground walking. (**Bottom**) Corresponding vertical displacement trajectories over time, with maximum stride heights ($d_{1},d_{2},d_{3}$) extracted for each clearance attempt. Successful clearance validation requires that calculated maximum stride height exceeds obstacle height ($d_{i}>h_{i}$) for each trial.

The second experiment assessed the system’s ability to measure foot clearance during obstacle negotiation. Participants were instructed to step over boxes of three different heights ($h_{1},h_{2},h_{3}$) placed on level ground, as shown in Figure 7. After each clearance attempt, the maximum stride height was extracted from the reconstructed trajectory. The validation criterion is that the calculated maximum stride height must exceed the known obstacle height ($d_{i}>h_{i}$), confirming that the calculated trajectory accurately reflects the clearance margin achieved by the participant. This experiment demonstrates whether the real-time height calculation provides sufficient accuracy to quantify foot clearance heights, a key requirement for assessing tripping risk and validating safe obstacle negotiation.

### 3.5. Statistical Analysis

Statistical analyses were performed using SPSS v29 (SPSS Inc., Chicago, IL, USA) with a significance level of α=0.05 [17,18]. Descriptive statistics are reported as mean ± standard deviation. Data normality was assessed via the Shapiro-Wilk test. To validate the height reconstruction accuracy, calculated total height changes were compared against architectural truth using paired *t*-tests. One-sample *t*-tests verified that obstacle clearances significantly exceeded object heights. Kinematic differences across multiple activities were evaluated using repeated-measures ANOVA with Bonferroni-corrected post hoc analysis. For non-normally distributed data, such as specific stair metrics, Wilcoxon signed-rank tests were employed. Effect sizes are reported as Cohen’s *d* or partial eta-squared ($\eta^{2}$). Bland–Altman analysis was additionally performed to assess agreement between calculated and architectural height values, reporting bias and 95% limits of agreement based on per-subject means for each activity.

## 4. Results

### 4.1. Heel Strike Velocity Error Calculation

**Figure 8:**
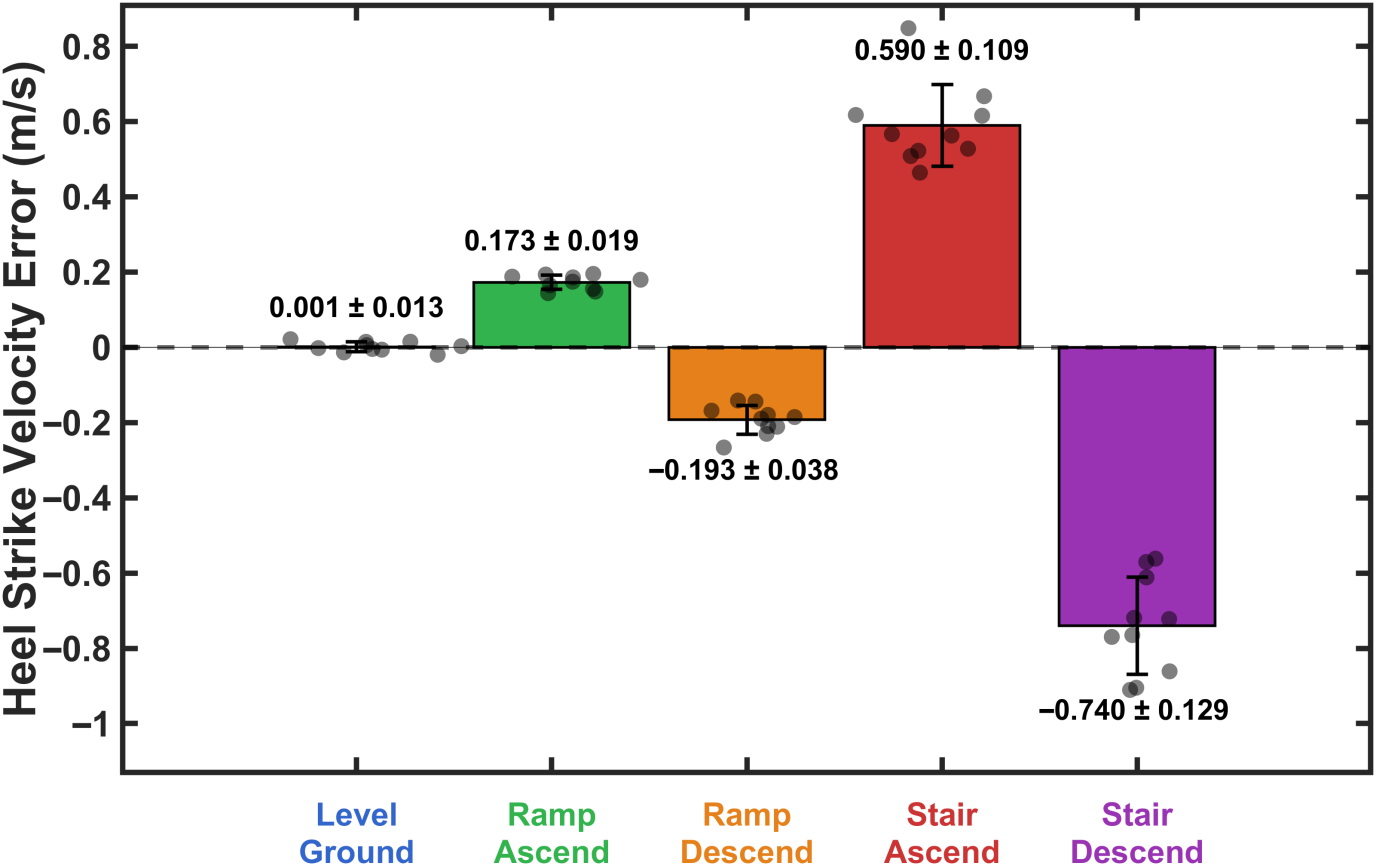
Mean heel strike velocity error ($v_{error,hs}$) across activities for the training group (N=10). Error bars represent standard error. All pairwise comparisons were significant (p<0.001).

The heel strike velocity error ($v_{error,hs}$) was calculated using data from the training group of participants. These results were then used to determine adaptive thresholds for real-time activity classification. Descriptive statistics for heel strike velocity error across locomotion activities are presented in Figure 8. heel strike velocity error was near zero during level walking (M=0.001±0.013), positive during ascending activities (ramp: M=0.173±0.019; stair: M=0.590±0.109), and negative during descending activities (ramp: M=−0.193±0.038; stair: M=−0.740±0.129). The Shapiro-Wilk test confirmed normal distribution of data for all activities (all p>0.05). A one-way repeated measures ANOVA revealed a significant main effect of activity on heel strike velocity error, F(4,36)=343.87, p<0.001, $\eta^{2}=0.974$. Bonferroni-corrected pairwise comparisons revealed significant differences between all activity pairs (all p<0.001). Level walking differed from all inclined/declined activities (all d>5.5). Within ascending activities, stairs showed greater positive bias than ramps (mean difference =0.417 m/s, p<0.001, d=3.97). Within descending activities, stairs exhibited greater negative bias than ramps (mean difference =−0.547 m/s, p<0.001, d=−4.68). All effect sizes were large [76], demonstrating substantial practical differences across activities.

To examine whether the magnitude of heel strike velocity error differed between ascending and descending activities, paired *t*-tests were conducted for both activity types. For ramp activities, the magnitude of error did not differ significantly between ascent and descent (p=0.074). In contrast, stair descent produced significantly greater error magnitude compared with stair ascent (p<0.001).

**Figure 9:**
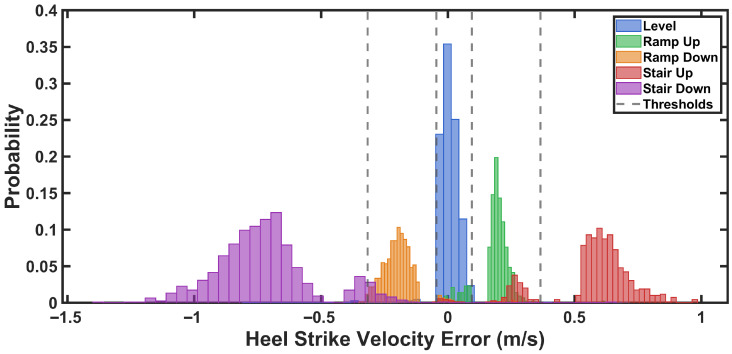
Probability distributions of heel strike velocity error ($v_{error,hs}$) for each activity. Clear separation between distributions enabled activity classification.

Figure 9 shows the distribution of heel strike velocity error for each activity from the training group data. The probability histograms demonstrate separation between all five locomotion activities, with level walking centered near zero, ascending activities exhibiting positive bias, and descending activities showing negative error.

### 4.2. Model Validation Analysis

#### 4.2.1. The Cumulative Height Experiment Results

**Figure 10:**
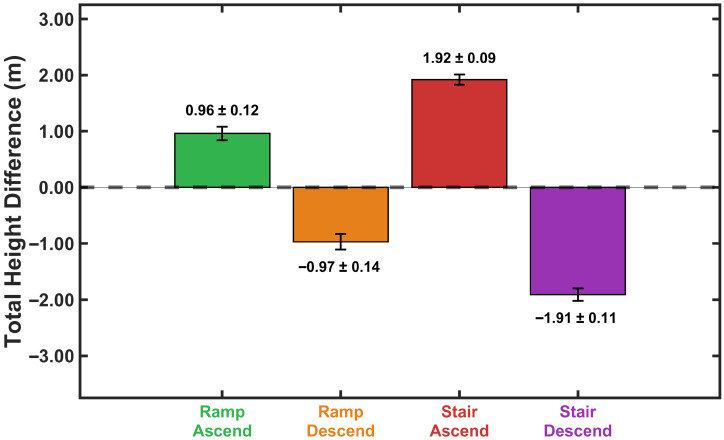
The calculated total height differences for ramp and stair activities in both ascent and descent directions as the validation for the cumulative height experiment. The actual measured dimensions were 0.96 m for the ramp and 1.921 m for the validation staircase (14 steps of 0.137 m rise each).

Cumulative height differences across complete ramp and stair traversals (Section 3.4.1) were compared against surveyed environmental dimensions using data from the testing group. Figure 10 presents measured total height differences for both ascent and descent directions. Actual dimensions were 0.96 m for the ramp and 1.921 m for the validation staircase (14 steps of 0.137 m rise each, 14×0.137m≈1.921m). One-sample *t*-tests assessed whether calculated heights differed significantly from true values. As shown in Table 1, none of the measured heights differed significantly from the actual environmental dimensions (all p>0.05), with a mean absolute error of 0.42% across all conditions. For ramp traversal, the model predicted heights of 0.96±0.12 m for ascent (error =0.0%, p=1.00) and 0.97±0.14 m for descent (error =1.04%, p=0.32). Stair traversal predictions were 1.92±0.09 m for ascent (error =0.05%, p=0.92) and 1.91±0.11 m for descent (error =0.57%, p=0.28). The 95% confidence intervals for all measurements encompassed the true values, further confirming the model’s accuracy.

Bland–Altman analysis was additionally performed and results are reported in Table 1, demonstrating small systematic biases with no consistent overestimation or underestimation pattern across activities. These results demonstrate that the proposed ZHC method eliminates cumulative drift in vertical position estimation during extended real-time operation, maintaining consistent height accuracy regardless of direction of travel.

#### 4.2.2. The Obstacle Clearance Experiment Results

**Figure 11:**
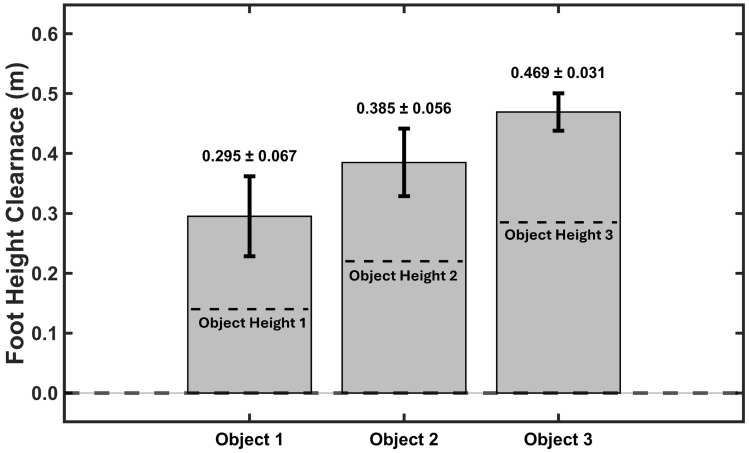
Mean maximum foot clearance heights for three obstacle heights. Error bars show standard deviation. All clearances significantly exceeded obstacle heights (all p<0.001).

Real-time obstacle clearance was validated using three boxes of different heights (14 cm, 22 cm, and 28.5 cm) positioned in the walking path. Figure 11 shows the maximum foot clearance heights measured during the obstacle clearance experiment, as mentioned in Section 3.4.2 with step-over maneuvers. The model successfully detected clearance for all objects, with measured heights of 0.295±0.067 m, 0.385±0.056 m, and 0.469±0.031 m for Objects 1, 2, and 3, respectively. One-sample *t*-tests confirmed that all clearances significantly exceeded box heights (all p<0.001), with safety margins ranging from 15.5 to 18.4 cm above obstacles, demonstrating adaptive clearance behavior. These results can show the model’s capability for real-time obstacle clearance monitoring.

### 4.3. Foot Trajectory Reconstruction Across Activities Results

**Figure 12:**
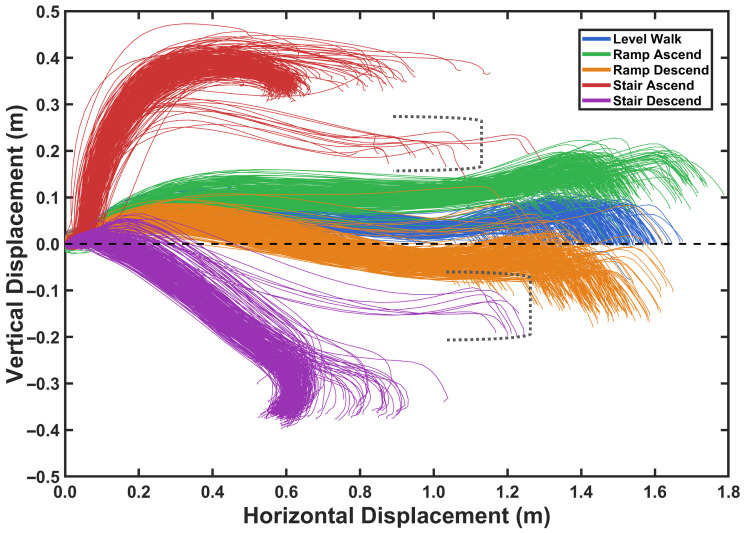
Reconstructed foot trajectories for five locomotion activities using the IMU-based kinematic model. Solid lines indicate steady-state strides, while dashed boxes identify transition strides occurring at staircase entry/exit points.

Using the kinematic model, foot trajectories showing horizontal and vertical displacement were reconstructed for each activity and segmented by stride, as depicted in Figure 12. The figure displays 200 randomly selected strides for each activity type. The trajectories demonstrate spatial separation between different locomotion activities, facilitating activity classification.

Using the kinematic model, foot trajectories showing horizontal and vertical displacement were reconstructed for each activity and segmented by stride, as depicted in Figure 12. Prior to visualization, terminal incomplete strides at the end of each recording session were excluded, as the absence of a second ZVP prevents solution of the constraint system, and the initial calibration strides of each trial were excluded from classification assessment as they were reserved for baseline parameter estimation. Given the remaining data volume, 200 strides per activity are randomly drawn from the retained dataset for visualization clarity. The trajectories demonstrate spatial separation between different locomotion activities, facilitating activity classification.

A notable characteristic of stair ascent and descent is the presence of two distinct trajectory groups. The first group, comprising the majority of strides, corresponds to steady-state negotiation where the foot traverses a full stride height, typically twice the individual step height, resulting in larger vertical displacement magnitudes. The second group, highlighted in the dashed-line boxes, represents transition strides at the entry or exit of the staircase, where the foot negotiates only a single step height, producing notably smaller vertical displacement magnitudes. This distinction reflects the difference between continuous stair climbing and the initial or final step of a staircase traversal, in which only half of the typical vertical excursion is required.

**Figure 13:**
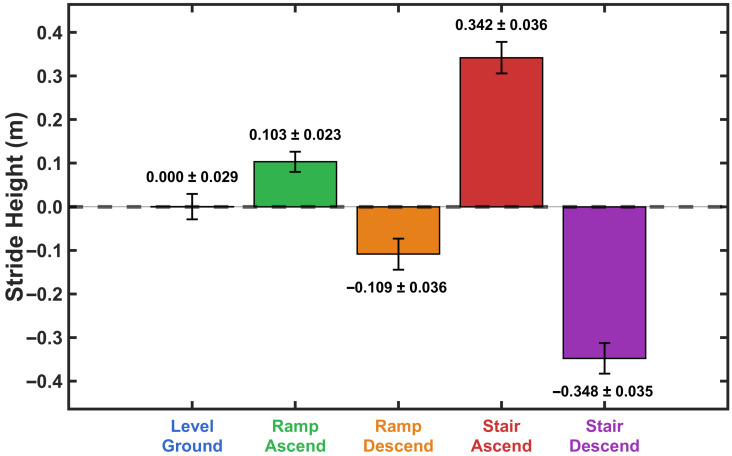
Mean stride height by activity with directional information (ascent positive, descent negative). Error bars represent standard deviation.

Figure 13 presents the mean stride height for each locomotion activity, demonstrating the model’s accuracy in reconstructing vertical foot displacement. Level walking exhibited a mean stride height of 0.000±0.029 m, effectively at ground level with minimal vertical clearance. Stair activities demonstrated the greatest vertical displacement, with stair ascent averaging 0.342±0.036 m and stair descent at −0.348±0.035 m. These measured values correspond closely to the expected stride height of the main staircase, where during steady-state negotiation each foot covers two steps per stride, yielding an expected stride height of 2×0.17m=0.34 m rather than the individual step height of 0.17 m. The measured stride heights therefore reflect this two-step-per-stride biomechanical pattern, with deviations of only 0.002 m (0.6%) for ascent and 0.008 m (2.4%) for descent. This analysis refers exclusively to the main staircase (step rise = 0.17 m) and is distinct from the validation staircase (step rise = 0.137 m) used for cumulative height assessment in Section 3.4.1. A one-sample *t*-test confirmed no significant difference between the measured stair ascent height and the actual step height (p=0.58), validating the accuracy of the proposed kinematic model with ZHC correction. Similarly, level walking showed no significant deviation from true ground level (p=1.00).

### 4.4. Reconstructed Trajectory in a Loop Results

**Figure 14:**
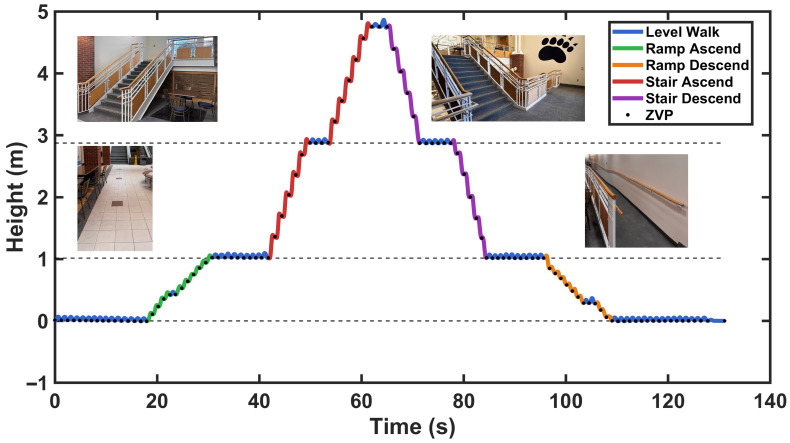
Continuous foot height trajectory across all locomotion activities with zero-velocity points (ZVP) marked as black dots. Inset images show the actual experimental activity, including level ground, ramp, and stairs.

Beyond individual stride analysis, the reconstructed foot trajectory was examined across a complete walking loop encompassing all activity types. Figure 14 presents the continuous height trajectory as a participant traversed the experimental environment, which included level walking, ramp negotiation, and stair climbing in sequence within a single loop. The images in Figure 14 show the actual experimental environment: flat ground for level walking, an inclined ramp with a plateau midpoint, stairs with an intermediate landing, and returning to the starting location to complete the loop.

### 4.5. Toe Trajectory Reconstruction

**Figure 15:**
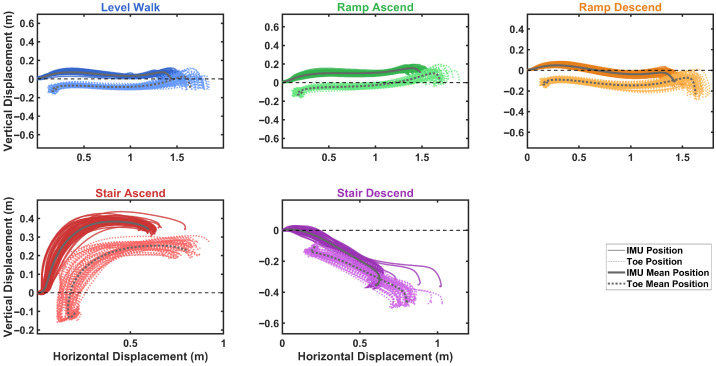
IMU (solid) and toe (dotted) position trajectories for each activity. Thick lines show mean trajectories. Toe positions calculated using rigid body transformation from IMU data.

As described in Section 2.5, toe position tracking is critical for assessing safe clearance during stair climbing and obstacle avoidance. Figure 15 presents the comparison between IMU-mounted (midfoot) and calculated toe trajectories across all five activities, aggregated from all participants in the testing group. The solid lines represent IMU position trajectories, while dotted lines show the corresponding toe positions derived through rigid body transformation. A consistent spatial offset is observed between the two trajectories, with the toe exhibiting more pronounced curved paths, particularly evident in the swing phase. This enhanced curvature at the toe reflects its position at the distal end of the rigid foot segment, where rotational motion about the ankle and metatarsophalangeal joints produces larger displacement arcs compared with the midfoot IMU location.

The mean trajectories in Figure 15 demonstrate consistent patterns within each activity type despite inter-subject variability, with the vertical separation between IMU and toe positions reflecting the rigid-body offset, which is largest during maximum plantar flexion at toe-off. Similar to the IMU stride height analysis in Figure 13, stride height was computed at the toe position for each activity, yielding −0.000±0.012 m for level walking, 0.105±0.020 m for ramp ascent, −0.105±0.026 m for ramp descent, 0.339±0.021 m for stair ascent, and −0.342±0.047 m for stair descent. Paired *t*-tests revealed no significant difference between IMU and toe stride heights across any activity (all p>0.5), supporting the validity of the rigid-body assumption for toe position estimation.

**Figure 16:**
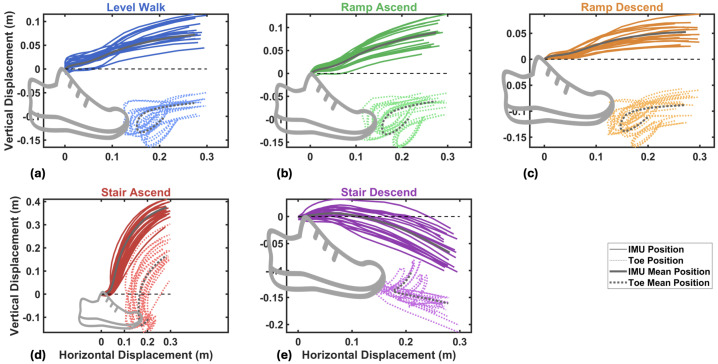
Zoomed view of the stride initiation phase across five activities: (**a**) level walking, (**b**) ramp ascent, (**c**) ramp descent, (**d**) stair ascent, and (**e**) stair descent. IMU (solid) and toe (dotted) trajectories are shown with thick lines indicating mean across participants. The dashed horizontal line represents ground level. Non-stair activities (**a**–**c**) exhibit curved toe-off trajectories from ankle rotation, while stair ascent (**d**) shows steep upward toe displacement and stair descent (**e**) shows controlled downward motion.

Figure 16 provides detailed views of the stride initiation phase (0–0.3 m horizontal displacement), where the biomechanical differences between IMU and toe motion are most apparent. During non-stair activities, level walking (a), ramp ascent (b), and ramp descent (c), the toe-off phase exhibits characteristic curved upward trajectories as the foot rotates about the ankle joint to generate forward propulsion. This rotation is visible in the toe trajectories but attenuated in the IMU position, reflecting the natural gait pattern where the foot pivots during push-off. In contrast, stair ascent (d) shows steep upward toe displacement to clear the step edge from the very first moments of the stride, while stair descent (e) initiates with a controlled downward toe trajectory below ground level, consistent with the foot being lowered onto the next step. The mean trajectories (thick solid and dashed lines) demonstrate consistent patterns within each activity type despite inter-subject variability. The vertical separation between IMU and toe positions reflects the rigid-body offset between the dorsal IMU mounting location and the toe tip, which varies with foot angle and is largest during maximum plantar flexion at toe-off.

### 4.6. Real-Time Activity Classification Accuracy

**Figure 17:**
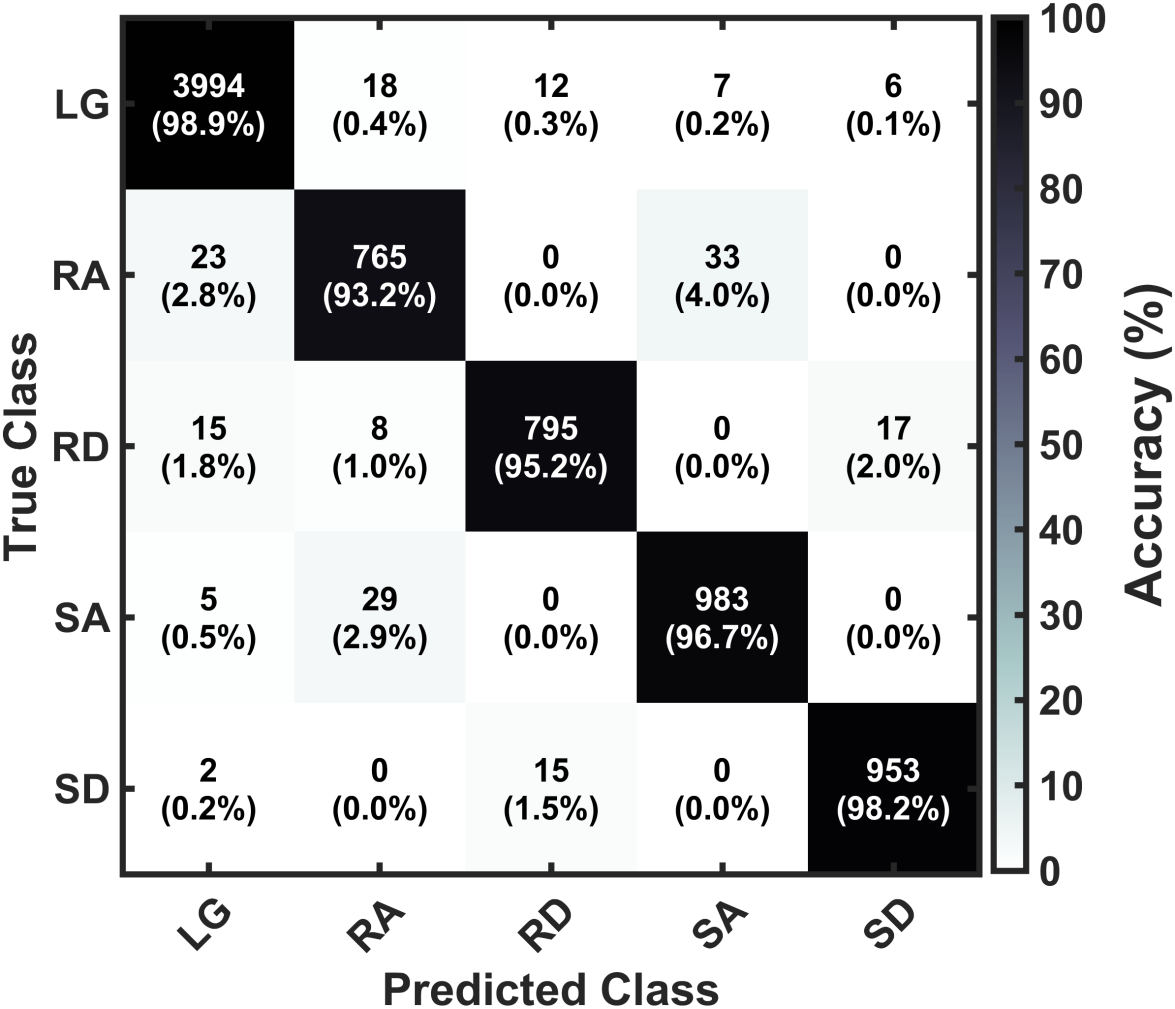
Confusion matrix for activity classification (n=7677 strides, 96.08% accuracy). LG: Level Walk, RA: Ramp Ascend, RD: Ramp Descend, SA: Stair Ascend, SD: Stair Descend.

To evaluate the real-time activity classification capability of the proposed system, stride-level predictions were analyzed across all five locomotion activities using data from the second participant group. Figure 17 presents the confusion matrix for the five-class activity classification task, evaluated on 7677 strides (LG: 4037; RA: 821; RD: 835; SA: 1017; SD: 970). The system achieved an overall accuracy of 96.08%, demonstrating discrimination across all activity types. The diagonal dominance of the confusion matrix indicates strong class separation, with the majority of strides correctly classified for each activity.

**Figure 18:**
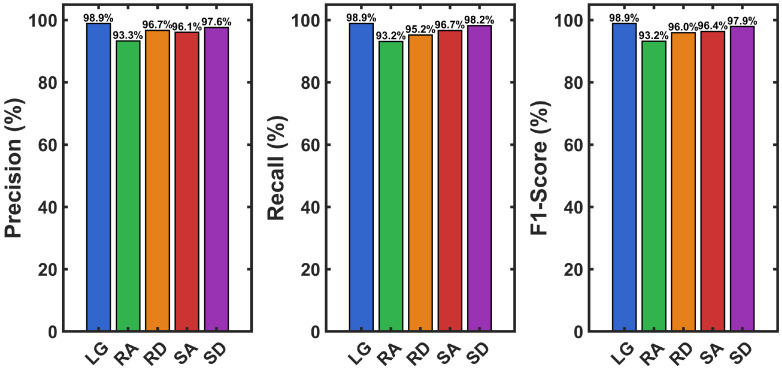
Precision, recall, and F1-score for each activity. All metrics exceed 90.7%, with a mean F1-score of 96.5%.

Activity-specific performance metrics are illustrated in Figure 18. Level walking demonstrated the highest performance with 98.9% recall, correctly identifying 3994 of 4037 strides, with a precision of 98.9% and F1-score of 98.9%. Stair descent showed 98.2% recall and 97.9% precision (F1-score: 98.2%), while stair ascent achieved 96.7% recall and 97.6% precision (F1-score: 96.4%). Ramp descent exhibited 95.2% recall and 96.1% precision (F1-score: 96.0%), while ramp ascent showed 93.2% recall and 96.7% precision (F1-score: 93.2%). The primary misclassifications for ramp ascent were 23 strides (2.8%) classified as level walking and 33 strides (4.0%) as stair ascent.

Analysis of misclassification patterns revealed that most errors occurred between activities with similar biomechanical characteristics. The largest confusion was between ramp ascent and stair ascent (33 strides, 4.0% of RA), which share elevated stride patterns. Similarly, ramp ascent was occasionally confused with level walking (23 strides, 2.8% of RA), likely during transition phases or low-incline sections where vertical displacement was minimal. Notably, there was confusion between ascent and descent directions within the same activity type, indicating that the calculated features effectively capture directional movement patterns. The mean precision, recall, and F1-score across all activities were 96.7%, 96.4%, and 96.5%, respectively, demonstrating balanced and consistent performance.

### 4.7. Stride Metrics Analysis Across Activities

**Figure 19:**
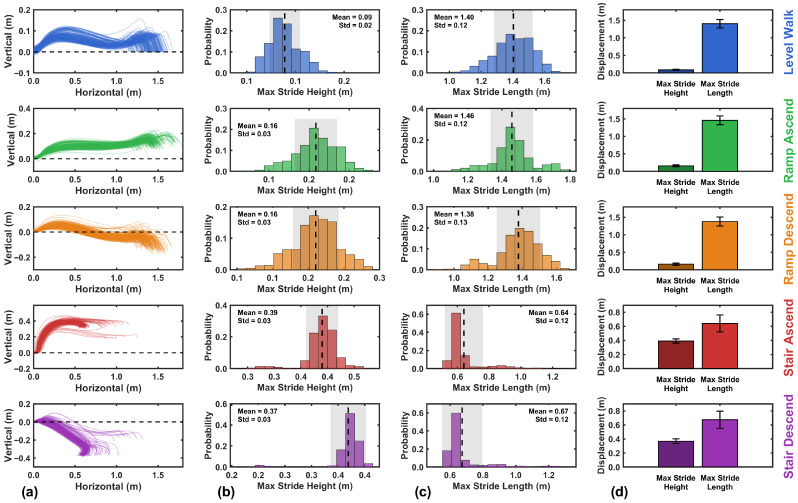
Activity-specific stride metrics showing clear separation between activity types. (**a**) Trajectory profiles, (**b**) maximum stride height distributions, (**c**) maximum stride length distributions, and (**d**) mean values with standard deviations. Dashed vertical lines indicate mean values.

To further characterize locomotion patterns, maximum stride height and stride length were analyzed for each activity (Figure 19). Stair ascent and descent exhibited notably narrower distributions compared with level walking and ramp activities, reflecting the biomechanical constraints imposed by fixed stair geometry. Level walking, ramp ascent, and ramp descent demonstrated broader distributions, indicating greater flexibility in stride adaptation.

Maximum stride height increased progressively with activity demand. Level walking showed the lowest values (0.09±0.02 m), ramp activities fell intermediate (0.16 m for both ascent and descent), and stair activities showed the highest clearance (ascent: 0.39±0.05 m; descent: 0.37±0.03 m). Notably, stair stride heights exceed the actual step rise, reflecting the additional foot clearance margin required during swing phase to prevent tripping. A one-way ANOVA confirmed significant differences across activities (p<0.001), with post hoc Tukey tests showing stair activities differed significantly from all other activity types (p<0.001).

Maximum stride length remained consistent across non-stair activities (level walking: 1.40 m; ramp ascent: 1.46 m; ramp descent: 1.38 m), suggesting horizontal progression is largely unaffected by elevation changes. Stair activities showed significantly shorter stride lengths (ascent: 0.64 m; descent: 0.67 m), constrained by stair tread depth.

Comparing stair ascent and descent, stair descent produced 6.3% greater maximum stride height than ascent (0.353±0.035 m vs. 0.332±0.036 m; Wilcoxon signed-rank test, p=0.001), reflecting increased toe clearance requirements when moving downward. In contrast, stride length showed no significant difference between directions (p=0.12), indicating horizontal progression remains constrained by stair geometry regardless of direction.

## 5. Discussion

### 5.1. Heel Strike Velocity Error Analysis

The statistically significant differences in heel strike velocity error across activities demonstrate its validity as a criterion for activity recognition. This parameter serves not only to distinguish level walking from non-level activities, but also to differentiate between ramp and stair locomotion and their respective directions. The sign of the bias indicates movement direction, with positive values corresponding to ascending activities and negative values to descending activities. The magnitude of heel strike velocity error was greater during ramp descent compared with ramp ascent, though this difference approached but did not reach statistical significance (p=0.072), suggesting a moderately increased heel strike impact during downhill ramp walking. In contrast, stair descent exhibited significantly larger bias magnitude than stair ascent (p<0.001), indicating a substantially greater heel strike impact during downward stair negotiation. These findings demonstrate that heel strike velocity error magnitude directly reflects the mechanical impact characteristics of heel contact during different locomotion tasks. The elevated impact forces during descent activities, particularly stair negotiation, may have important implications for fall risk assessment and prevention strategies.

### 5.2. Model Validations Analysis

#### 5.2.1. The Cumulative Height Experiment

The first validation experiment assessed cumulative vertical displacement accuracy. As shown in Figure 10 and Table 1, measured heights matched the actual dimensions with a mean absolute error of 0.42% across all conditions. For ramp negotiation, the model estimated 0.96 m (ascent) and 0.97 m (descent) against the actual 0.96 m elevation, with narrow confidence intervals (±0.12–0.14 m) and non-significant deviations (p>0.32). This accuracy persisted despite an intermediate-level platform, demonstrating that ZHC correction maintains height reference when brief level segments interrupt continuous ascent or descent. Stair negotiation estimates of 1.92 m (ascent) and 1.91 m (descent) aligned with the actual six-step height of 1.92 m, with tighter confidence intervals (±0.09–0.11 m) likely reflecting the constrained geometry of stair negotiation.

The marginally higher height error observed for ramps compared with stairs may appear counterintuitive given the greater impact dynamics of stair gait. However, ramp ascent and ramp descent achieved the lowest classification accuracies (Figure 17) among all activities (93.2% and 95.2% respectively), owing to their biomechanical similarity to level walking. When a ramp stride is misclassified as level walking, the full constraint system enforcing zero height change is incorrectly applied, directly introducing height error. Stair ascent and stair descent achieved higher accuracies (96.7% and 98.2%, respectively), so the correct reconstruction model is applied more consistently, yielding lower cumulative height error despite higher impact dynamics.

Comparable accuracy across ascent and descent directions (maximum error 1.04%) indicates bidirectional reliability essential for real-world applications. Traditional ZUPT-based methods would accumulate substantial drift over these extended traversals, particularly during multi-step stair sequences, highlighting the ZHC approach’s advantage for maintaining long-term vertical position accuracy.

#### 5.2.2. The Obstacle Clearance Experiment

The main objective of this experiment was to demonstrate that the natural safety margin during obstacle crossing is an order of magnitude larger than the variability in vertical displacement estimation using our method. We assessed foot clearance detection during obstacle avoidance, quantifying heights for three obstacles (14 cm, 22 cm, 28.5 cm) as 0.295 m, 0.385 m, and 0.469 m, respectively (Figure 11). All measured clearances significantly exceeded actual obstacle heights (p<0.001), with safety margins ranging from 15.5 to 18.4 cm. These margins reflect natural adaptive gait behavior, where individuals elevate their feet substantially above obstacles to reduce tripping risk.

The system successfully detected clearance margins above all three obstacle heights, confirming that the real-time height estimation provides sufficient resolution to distinguish safe from insufficient clearance. This capability could enable haptic or auditory alerts between strides when estimated clearance falls below a predefined safety threshold, providing an inter-stride feedback signal to modulate foot clearance behavior in the subsequent step.

### 5.3. Foot Trajectory Reconstruction Across Activities

The reconstructed foot trajectories, following heel strike velocity error calculation and activity detection, demonstrated distinct patterns for each activity type. Notably, the model captured transitional stair negotiation cases, such as entering or exiting the stair environment, which involve traversing a single step height rather than the typical two-step pattern of continuous stair climbing. As shown in Figure 12, these single-step transitions exhibited trajectories that closely resembled ramp ascent or descent patterns. This can highlight the model’s ability to distinguish between normal stair negotiation and its transition moment. The validation results in Figure 13 demonstrate accurate stride height estimation. Level walking stride height of 0.000±0.029 m confirms the effectiveness of ZHC correction in maintaining ground reference. The measured stair ascent stride height (0.342 m) matched the expected value of twice the individual step rise (2×0.17 m =0.34 m) with only 0.6%. Note that the stride height of ∼0.34 m reflects two steps of 0.17 m per stride on the main staircase, not the individual step height. The slightly larger deviation for stair descent (2.4%) likely reflects the inclusion of transition strides at staircase entry and exit points, which involve single-step traversal rather than full two-step rise. The ability to quantify these stride parameters in real-time enables assessment of foot clearance adequacy and stride variability, which could inform adaptive feedback strategies.

The marginally higher errors observed for non-level activities (0.05–1.04%) compared with level walking (0.00%) are consistent with the reduced 3×3 constraint set, where $v_{\mathrm{error},\mathrm{hs}}$ cannot be explicitly estimated and is instead absorbed into $a_{\mathrm{bias}}$, introducing a small residual error that is absent in the full 4×4 level-walking system.

It should also be noted that both the staircase and ramp included short intermediate level-walking plateaus, during which the ZHC constraint re-engages and corrects any residual vertical drift, providing natural drift reset points that further bound cumulative height error.

### 5.4. Reconstructed Trajectory in a Loop

As shown in Figure 14, the foot height during level walking segments remains at a consistent reference level throughout the trial, before ascending the stairs and ramp, at intermediate platforms, and after returning to ground level. This height consistency at ZVP on level ground validates the proposed method’s ability to maintain accurate vertical position estimation. This consistency could not be achieved using traditional ZUPT-based methods due to cumulative drift in vertical position estimation [33,63]. The proposed Zero Height Clearance (ZHC) method effectively eliminates this drift, maintaining an accurate height reference across the entire walking sequence without requiring external position resets.

The model demonstrates sensitivity to activity changes, identifying even brief level walking segments at intermediate platforms. The experimental environment included rest areas at both the middle of the ramp and stair configurations, where participants briefly walked on flat ground. The model classified these short plateau regions as level walking, demonstrating its ability to capture transient activity transitions. Notably, the ramp plateau appears shorter in duration compared with the stair plateau, accurately reflecting the actual geometry of the test environment. This real-time adaptability to changing activity conditions is critical for fall prevention applications, where timely detection of environmental transitions enables appropriate adjustment of haptic feedback or alert parameters to match the current locomotion context.

### 5.5. Toe Position Assessment

The rigid body transformation could extend the kinematic model from the IMU to the toe, the most relevant landmark for clearance assessment. As shown in Figure 15 and Figure 16, the toe exhibits more pronounced curved paths during the swing phase, particularly at toe-off, due to larger displacement arcs produced by ankle rotation at this distal position.

The vertical separation between IMU and toe positions (5–15 cm) varies with foot orientation, reaching a maximum during toe-off at peak plantar flexion. This highlights why toe position provides more relevant clearance information than IMU position alone. The distinct patterns in Figure 16 demonstrate activity-specific foot motion: level walking and ramp activities show curved toe-off trajectories for forward propulsion, while stair ascent exhibits immediate upward toe trajectories to clear steps, and stair descent shows controlled downward motion. The consistency of mean trajectories across participants indicates the transformation maintains accuracy across different foot sizes and gait patterns. Beyond clearance monitoring, toe trajectory information could assess ankle mobility during toe-off or detect changes in minimum clearance that may reflect fatigue or declining strength. Finally, real-time toe position serves as a metric for tripping risk assessment in gait training applications. Furthermore, the absence of significant differences between IMU and toe stride heights across all activities confirms that the kinematic accuracy validated at the IMU location transfers to the toe under the rigid-body assumption, providing the validation of the toe position estimates.

### 5.6. Real-Time Activity Classification Performance

The activity recognition system achieved 96.08% overall accuracy across five locomotion activities, demonstrating real-time capability to distinguish walking environments using heel strike velocity error and kinematic features. Level walking exhibited the highest performance (98.9% F1-score) due to its minimal vertical displacement and consistent stride patterns. Stair activities achieved strong performance (ascent: 96.4%; descent: 98.2%), with descent’s higher accuracy likely reflecting more pronounced heel strike impact and greater vertical clearance that provide clearer discriminative features.

Ramp activities showed comparatively lower but still adequate performance (ramp ascent: 93.2% F1-score; ramp descent: 96.0% F1-score). The primary misclassifications for ramp ascent were confusion with stair ascent (4.0%) and level walking (2.8%). The confusion between ramp ascent and stair ascent is expected given their shared characteristics of upward movement and elevated stride height. The confusion with level walking likely occurs during transition phases at ramp entry and exit points, or on low-incline sections where vertical displacement approaches that of level ground. The presence of intermediate-level platforms within the ramp section may also contribute to these misclassifications during brief plateau segments.

This transition-related misclassification pattern extends across all activity pairs in proportion to their biomechanical similarity, including ramp descent misclassified as stair descent (17 strides, 2.0%) and stair ascent misclassified as ramp ascent (29 strides, 2.9%), consistent with findings reported by Wang et al. [33] and our prior work [48], confirming that transition-related misclassification is a known inherent challenge in stride-by-stride terrain classification rather than a specific limitation of the ZHC formulation.

The absence of confusion between ascent and descent directions within the same activity type validates the effectiveness of using signed heel strike velocity error as a directional indicator. The sign of this parameter (positive for ascent, negative for descent) provides clear directional information that prevents misclassification between opposing movement directions. The stride-by-stride independence of the ZHC framework ensures robustness at terrain transitions, since $v_{error,hs}$ is re-estimated freely at every stride boundary without memory of previous classifications, making any misclassification self-correcting within one stride.

### 5.7. Stride Metrics Analysis

The analysis of stride metrics demonstrates the model’s ability to extract biomechanically relevant parameters to assess foot clearance. The distribution characteristics in Figure 19 reveal how environmental constraints influence gait variability across activity types. The narrower distributions observed for stair activities than for level walking and ramp locomotion reflect the geometric constraints of stair negotiation, in which participants must conform to fixed step dimensions. Level walking and ramp activities exhibited broader distributions, indicating greater flexibility in stride adaptation.

The maximum stride height values for stair ascent (0.39 m) and descent (0.37 m) exceed the actual stair height due to the toe clearance margin required during the swing phase. The significantly greater maximum stride height during stair descent compared with ascent (p<0.001, Cohen’s d=−0.60) indicates increased vertical clearance requirements when moving downward, likely reflecting a cautious gait strategy to ensure safe foot placement.

Stride length remained consistent across level walking (1.40 m), ramp ascent (1.46 m), and ramp descent (1.38 m), suggesting that participants maintained similar horizontal progression despite elevation changes. The ramp inclination did not substantially alter stride length, indicating adaptation occurred primarily through vertical displacement. In contrast, stair activities showed shorter stride lengths (0.64–0.67 m), dictated by stair depth and vertical movement. The lack of difference between stair ascent and descent stride length (p=0.13) indicates horizontal progression remains constrained by stair geometry regardless of direction.

### 5.8. Comparison with Previous Methods

Table 2 summarizes existing IMU-based methods for foot height estimation and activity classification. The primary contribution of the proposed method is real-time, continuous vertical foot position estimation across multiple activity types using a single foot-mounted IMU deployed on a smartphone, a capability absent from all prior work. Activity classification serves as an enabling component of accurate height reconstruction rather than the primary objective.

Most existing methods address either trajectory estimation or activity recognition in isolation, and those that do estimate vertical position are limited to level walking and offline processing. Benoussaad et al. [77] estimated foot clearance during level walking using double integration with drift cancellation, reporting mean errors below 15% against motion capture, but without terrain classification or real-time capability. Jocham et al. [78] achieved an average vertical RMSE of 0.51 cm for foot position trajectories during level walking using foot-mounted IMUs, but were limited to level walking and offline processing. Fehr et al. [26] estimated whole-foot minimum clearance with 95% limits of agreement of ±8.1 mm against optical motion capture using a personalized 3D shoe geometry scan, but required offline processing and were limited to level walking.

Wang et al. [33], the most comparable prior work, achieved foot trajectory reconstruction and terrain classification (99.7% accuracy) across all five activity types using a single foot-mounted IMU, but their method operates entirely offline in MATLAB, precluding real-time deployment. Notably, this offline method still reported mean height errors of 0.95±0.37 cm on ramps and 1.27±1.22 cm on stairs per stride, comparable to the typical MFC magnitude of 1 cm [79], suggesting that precise per-stride MFC quantification is a fundamental challenge for single foot-mounted IMU approaches regardless of processing mode.

For activity classification, Gao et al. [80] achieved 98.5% accuracy across five terrain types including ramps, but without foot trajectory estimation. Kang et al. [81] classified five activities including ramps (94.9%) using a deep CNN, but required three leg-mounted IMUs with no trajectory output. Cheng et al. [82], Moura Coelho et al. [83], Sharafian et al. [48], and Zhou et al. [84] achieved high classification accuracy (98–99%) but none provide foot height estimation or ramp detection. Bhakta et al. [85] developed a real-time slope estimation system but required extensive instrumentation, limiting general applicability.

In contrast, the proposed method achieves real-time vertical foot position estimation with cumulative height errors below 1.1 cm across ramp and stair negotiation, and a mean absolute error of 0.42%, while simultaneously classifying five locomotion activities with 96.08% accuracy at 110 ms latency, all using a single foot-mounted IMU on an Android smartphone.

A direct quantitative comparison of vertical accuracy requires careful interpretation due to differing error bases across studies (Table 2). Jocham et al. [78] reported 0.51 cm RMSE and Fehr et al. [26] achieved ±8.1 mm per-stride accuracy, both restricted to level walking with offline processing. Wang et al. [33], the most comparable prior work, reported per-stride height errors of 0.95±0.37 cm on ramps and 1.27±1.22 cm on stairs using an offline MATLAB implementation. The proposed method achieves cumulative height errors below 1.1 cm (0.42% MAE) across stair and ramp traversals, comparable to Wang et al., while operating in real-time on a smartphone. It should be noted that cumulative and per-stride errors are not directly equivalent metrics, and both are relevant to different clinical applications. The primary advantage of the proposed method over all prior work therefore lies in real-time smartphone deployment across five locomotion activity types using a single foot-mounted IMU, rather than superior per-stride vertical accuracy.

**Table 2:** Comparison of IMU-based foot height estimation and activity classification methods with the proposed system. Height error metrics differ in basis across methods: cumulative traversal error is reported for Wang et al. [33] and the proposed method; per-stride RMSE for Jocham et al. [78] and Fehr et al. [26]; and percentage error against motion capture for Benoussaad et al. [77] Direct numerical comparison across methods should account for these differing bases, as cumulative and per-stride errors are not equivalent metrics.

Study	Sensors	Hz	Activities	Method	Outputs	N	Height Error	Class. Acc.	Latency	Real-Time	Platform
Benoussaad et al. (2016) [77]	1 (foot)	–	LW	Double integration + drift cancel	Foot clearance	–	<15%	N/A	–	No	Offline
Gao et al. (2020) [80]	1 (foot)	–	LW, RA, RD, SA, SD	Elliptical boundary	Terrain	6	N/A	98.5%	–	Yes	–
Cheng et al. (2021) [82]	1 IMU, 1 FSR	–	LW, SA, SD, SI	ICF + KNN/SVM	HAR	3	N/A	99.4%	5 ms	Yes	Raspberry Pi
Kang et al. (2022) [81]	3 (leg)	200	LW, RA, RD, SA, SD	Deep CNN	Terrain	22	N/A	94.9%	–	Yes	–
Moura Coelho et al. (2022) [83]	1 (foot)	333	LW, SA, SD	CNN-LSTM	Terrain	10	N/A	98%	90 ms	Yes	–
Fehr et al. (2024) [26]	1 (foot) + 3D scan	128	LW	ZUPT + geometry	Foot height	3	±8.1 mm ^†^	N/A	–	No	Offline
Jocham et al. (2024) [78]	1 (foot)	–	LW	IMU fusion	Foot position	–	0.51 cm RMSE	N/A	–	No	Offline
Wang et al. (2024) [33]	1 (foot)	128	LW, RA, RD, SA, SD	HS velocity threshold	Traj., Terrain	6	0.03–1.27 cm/stride	99.7%	–	No	Offline
Bhakta et al. (2025) [85]	3 IMUs + encoders + loadcell	–	LW, RA, RD, jog	XGBoost	Slope, Speed	11	N/A	5.4% error	157 ms	Yes	Raspberry Pi 4
Zhou et al. (2025) [84]	1 (wrist)	–	LW, SA, SD, ST, SI, jog	DeepConv LSTM	HAR	–	N/A	98.2%	2000 ms	Yes	Arduino Nano
Sharafian et al. (2025) [48]	2 (thigh, foot)	60	LW, SA, SD, ST, SI	KNN (angles)	HAR	25	N/A	99%	52 ms	Yes	Android
**This study**	**1 (foot)**	**60**	**LW, RA, RD, SA, SD**	**ZHC + HS bias**	**Foot height, HAR**	**20**	**<1.1 cm (0.42% MAE)**	**96.1%**	**110 ms**	**Yes**	**Android**

**Abbreviation:** LW = Level Walking; RA/RD = Ramp Ascent/Descent; SA/SD = Stair Ascent/Descent; ST = Standing; SI = Sitting; HS = Heel Strike; ZHC = Zero Height Change; ICF = Instantaneous Characteristic Features; FSR = Force Sensitive Resistor; Traj. = Trajectory; HAR = Human Activity Recognition; MAE = Mean Absolute Error; RMSE = Root Mean Square Error. ^†^ 95% limits of agreement against optical motion capture.

A further limitation concerns vertical accuracy relative to the typical magnitude of MFC. Previous studies have reported MFC during level walking to be on the order of 1 cm [79], while the stride-level vertical variability of the proposed system (±0.029 m, Figure 13) is larger than this magnitude. This presents limitations for avoiding toe catch during normal level-ground walking, where precise sub-centimeter MFC quantification is required. However, the primary application of our method is obstacle crossing and stair negotiation training, where MFC is substantially greater, on the order of 10 cm or more, as demonstrated in Figure 11, and therefore well within the measurement capability of the proposed system. While cumulative height errors remain low (0.42% MAE), precise per-stride MFC quantification at the 1 cm scale remains challenging with the current implementation, and the system is better positioned for monitoring coarser metrics. At the current accuracy level, the system supports stride height estimation and cumulative stair and ramp height measurement [33] (sub-1.1 cm cumulative error, Table 1), real-time activity classification [48,80], and obstacle clearance detection where safety margins substantially exceed measurement uncertainty [22]. Applications requiring precise per-stride MFC quantification at the sub-centimeter scale [21,79] are not currently supported and represent an important direction for future work.

### 5.9. Limitations

This study had several limitations that should be considered when interpreting the results. A significant limitation of the present study is the absence of optical motion capture for trajectory validation. Motion capture would provide per-instant trajectory verification and direct validation of minimum foot clearance, which architectural ground truth cannot offer, and we acknowledge that low cumulative error does not guarantee per-instant trajectory accuracy. However, typical camera-based motion capture systems are ideal for treadmill walking or walking in a small capture volume, and are not feasible for long walking trails or walking on stairs or ramps in realistic daily walking scenarios. Furthermore, the reconstructed foot trajectories demonstrate activity-specific shapes consistent with established biomechanical patterns, providing qualitative support for the validity of the reconstruction framework. Future clinical validation should incorporate motion capture in controlled activity subsets to provide per-instant trajectory verification alongside the cumulative accuracy demonstrated here.

As discussed in Section 5.8, the system is not suited for precise per-stride MFC quantification at the sub-centimeter scale, but is better positioned for coarse metrics such as stride height, cumulative height estimation, and obstacle clearance detection where safety margins are substantially larger. Moreover, the 60 Hz sampling rate may limit temporal resolution for capturing fine-scale gait events. The minimum detectable vertical displacement at this sampling rate is approximately 1.37 mm, which can be sufficient for obstacle crossing and stair negotiation applications where MFC is substantially larger, but may limit sub-centimeter level-ground MFC quantification. Higher sampling frequencies could improve trajectory reconstruction and represent a direction for future work; however, vertical accuracy rather than temporal resolution is the primary bottleneck for precise MFC quantification in single foot-mounted IMU systems [33]. Additionally, no post-sensor filtering was applied to the acceleration signal beyond the Xsens DOT onboard processing pipeline. While the sensor’s internal low-pass filtering attenuates high-frequency electronic noise prior to data delivery, residual noise from heel strike dynamics may still propagate into double integration. Future work could explore selective filtering strategies to further reduce integration noise and improve trajectory reconstruction accuracy.

The participant sample was limited to 20 healthy younger adults (10 for training, 10 for testing), which represents a significant limitation in terms of transferability to the primary target population. Older adults, who are the primary target population for fall prevention applications, may exhibit slower gait speed, reduced foot clearance, altered heel strike dynamics, and greater stride variability compared with young healthy adults, all of which could affect ZVP detection reliability, classification accuracy, and kinematic reconstruction performance in ways not captured by the present validation. The present study establishes proof-of-concept performance in a controlled healthy population, and clinical validation with older adults and individuals with mobility impairments such as Parkinson’s disease, stroke, and peripheral neuropathy is a necessary and primary future direction before deployment in fall prevention applications.

The toe position calculation assumed a rigid foot segment, which does not fully capture the natural flexibility of the foot during gait. In reality, the foot undergoes metatarsophalangeal joint flexion during toe-off and may exhibit arch deformation under load. These deformations could introduce errors in toe position estimation, particularly during push-off phases where non-rigid motion is most pronounced.

Furthermore, the experimental environment, while including diverse activity types, was controlled and familiar to participants. Real-world environments present additional challenges, including irregular surfaces, varying stair dimensions, non-standard ramp angles, cluttered spaces, and unexpected obstacles. The model’s performance under these unconstrained conditions remains to be validated. Addressing these challenges will require more robust algorithmic techniques for ZVP detection and activity classification under complex real-world conditions, while preserving the real-time latency performance demonstrated in the present study.

The impact of prolonged use on measurement drift and the effects of different footwear types on kinematic accuracy were not investigated. Finally, the heel strike velocity error calculation and activity classification were developed and tested on the same experimental setup. External validation on independent datasets from different environments and populations is needed to confirm the model’s transferability and robustness.

Classification accuracy is reported at the stride level, and strides within the same participant are not independent observations. While the model was evaluated on a fully held-out testing group not involved in any stage of model development, stride-level metrics may overestimate generalization performance relative to subject-level evaluation, and future work should include subject-level cross-validation across larger and more diverse populations.

### 5.10. Future Directions

Several avenues for future research could address current limitations and extend system capabilities. Clinical validation with older adults and individuals with mobility impairments (Parkinson’s disease, stroke, peripheral neuropathy) is essential to assess performance in target fall prevention populations and enable development of condition-specific models. Enhanced foot modeling incorporating metatarsophalangeal joint motion and arch deformation, potentially using machine learning to estimate non-rigid deformation from midfoot IMU data, could improve toe position accuracy.

Real-world deployment in uncontrolled environments with irregular surfaces, unexpected perturbations, and naturalistic walking patterns remains a primary future direction. Addressing these challenges will require more robust algorithmic techniques for ZVP detection and activity classification under complex conditions, while preserving the real-time latency performance demonstrated in the present study. Long-term studies assessing measurement drift, battery life, user acceptance, and practical feasibility during daily activities will also be essential before clinical deployment. Expanding activity recognition to include turning, backward walking, sit-to-stand transitions, and pre-fall events (stumbles, balance perturbations) would enable more comprehensive monitoring and proactive intervention. Finally, with the ZHC method enabling real-time operation, integration with interventions such as haptic feedback, auditory alerts, or adaptive exoskeleton control could demonstrate clinical utility, with studies examining whether timely alerts reduce fall rates or improve mobility confidence in at-risk populations.

## 6. Conclusions

This study presented a real-time, single-IMU system for simultaneous foot height trajectory reconstruction and locomotion activity classification, deployed on a consumer Android smartphone. The Zero Height Change (ZHC) method eliminated cumulative vertical drift during level walking by enforcing biomechanical boundary conditions at stride boundaries, addressing a fundamental limitation of conventional ZUPT-based methods. By leveraging heel strike velocity error as a compact, physically meaningful feature, the system achieved adaptive activity classification across five locomotion activities without requiring multi-sensor configurations or offline processing. Validation results demonstrate an acceptable accuracy of the proposed framework. Cumulative height estimation across extended stair and ramp traversals yielded a mean absolute error of 0.42% against architectural ground truth, confirming long-term vertical position reliability. Obstacle clearance was successfully quantified across all tested heights, and the system achieved 96.08% overall activity classification accuracy with a latency of less than one gait cycle. Together, these contributions represent a meaningful advancement toward practical, wearable gait monitoring systems. The ability to simultaneously measure foot height and classify activity in real-time on a smartphone opens pathways for integration with haptic feedback, auditory alerts, and adaptive assistive devices, supporting broader applications in gait rehabilitation, clinical assessment, and fall prevention. Though at the current accuracy level, the system is suited for coarse foot clearance metrics and cumulative height changes, with precise per-stride MFC quantification at the sub-centimeter scale remaining a direction for future work.

## Figures and Tables

**Table 1:** Statistical validation of calculated total height difference in real-time against actual environmental dimensions. One-sample *t*-tests compare measured heights to actual values (ramp: 0.96 m; validation staircase: 1.921 m, comprising 14 steps of 0.137 m rise). Bland–Altman bias and 95% limits of agreement (LoA) are additionally reported based on per-subject means.

Activity	Measured (m)	Actual (m)	Error (%)	95% CI	*p*-Value	Significant?	Bias (m)	95% LoA (m)
Ramp Ascend	0.96±0.12	0.96	0.00	[0.94, 0.98]	1.000	No	+0.000	[−0.235, 0.235]
Ramp Descend	0.97±0.14	0.96	1.04	[0.94, 1.00]	0.321	No	+0.010	[−0.264, 0.284]
Stair Ascend	1.92±0.09	1.921	0.05	[1.91, 1.93]	0.921	No	−0.001	[−0.177, 0.175]
Stair Descend	1.91±0.11	1.921	0.57	[1.89, 1.93]	0.276	No	−0.011	[−0.227, 0.205]

**Figure A1:**
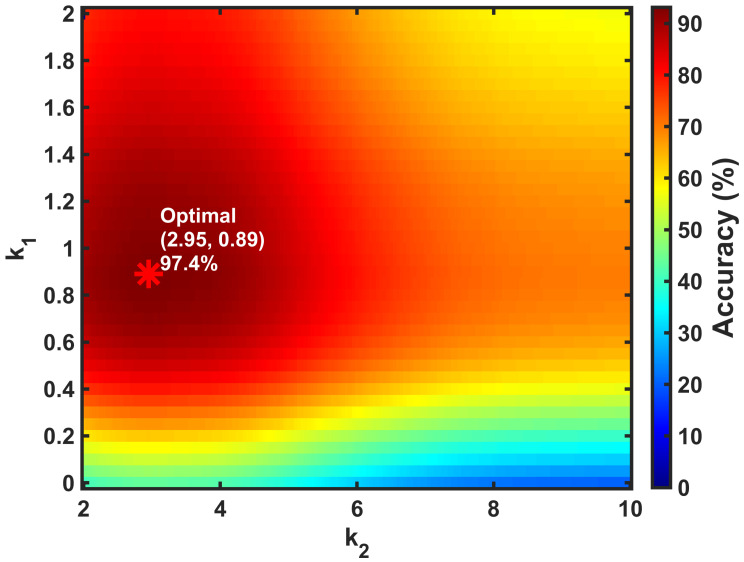
Heat map showing classification accuracy as a function of threshold multipliers $\left(k_{1},k_{2}\right)$ across all training data. The grid search optimization identifies the optimal parameter pair $\left(k_{1}^{*},k_{2}^{*}\right)=\left(0.89,2.95\right)$, achieving 97.4% classification accuracy. These optimized values, determined through offline processing of labeled training data, are subsequently deployed for real-time activity classification.

**Figure A2:**
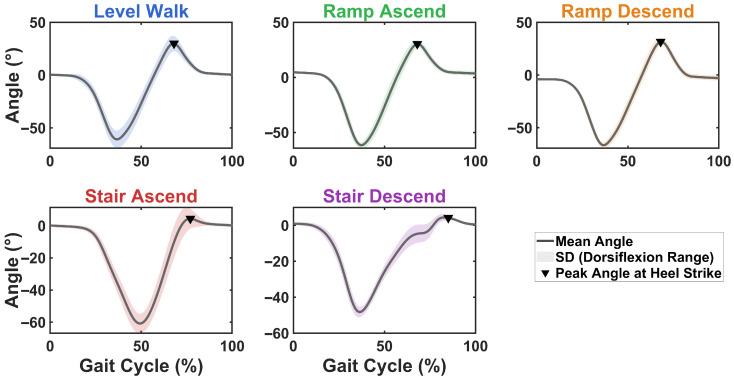
Representative foot pitch angle profiles across the normalized gait cycle for all five locomotion activities from one participant. The shaded region represents ±1 SD across strides. The filled triangle (▼) indicates the detected heel strike instant $k_{hs}$ corresponding to the maximum pitch angle within each stride.

**Figure A3:**
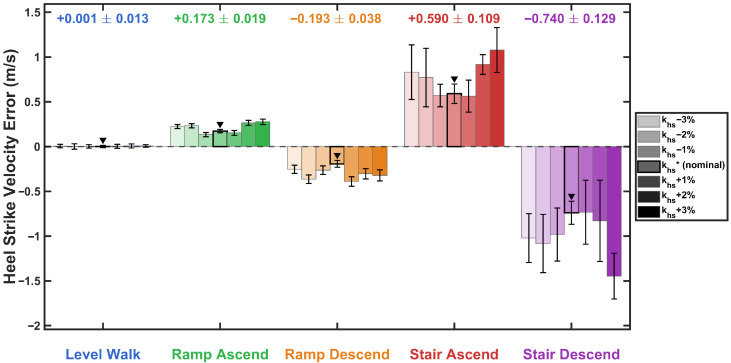
Sensitivity of heel strike velocity error ($v_{\mathrm{error},\mathrm{hs}}$) to perturbations in heel strike timing ($k_{hs}$) across all five locomotion activities. The filled triangle (▼) indicates the nominal (unperturbed) value. Error bars represent ±1 SD. The baseline mean ± SD for each activity is shown at the top of the figure.

**Figure A4:**
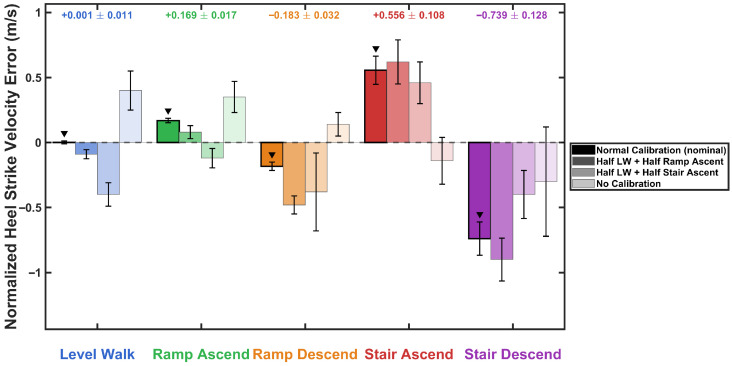
Normalized heel strike velocity error (${\hat{v}}_{error,hs}$) across five locomotion activities for four calibration scenarios (mean ± SD). Progressive calibration contamination shifts $\mu_{hs}$ positively, compressing inter-activity separation and degrading classification performance. Nominal values from S1 are annotated at the top of each activity group.

**Figure A5:**
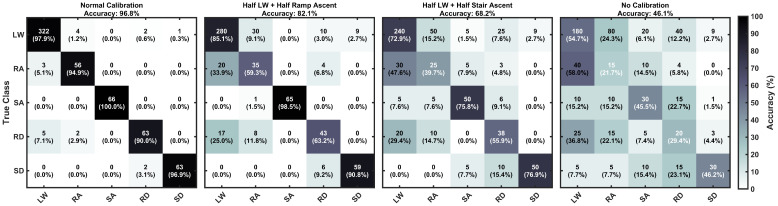
Confusion matrices for four calibration scenarios (rows = true class, columns = predicted class). Overall accuracy decreases progressively from S1 (Normal, 96.8%) to S2 (Half Ramp, 82.1%), S3 (Half Stair, 68.2%), and S4 (No Calibration, 46.1%).

**Figure A6:**
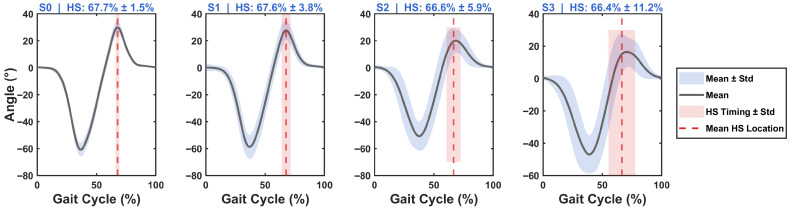
Sensitivity analysis of ZVP detection parameters. Mean foot pitch angle trajectory (dark line), standard deviation band (blue), and HS timing distribution (red dashed line ± shaded region) are shown for each scenario. S0: baseline ($T_{a}=15\;{m/s}^{2}$, $T_{\omega }=4\;\mathrm{rad}/s$, $W_{cap}=8$, $T_{max}=80\;\mathrm{ms}$); S1: $T_{a}$, $T_{\omega }$ increased by 100%; S2: $W_{cap}$ decreased by 100%; S3: $T_{max}$ decreased by 100%. S1 preserves baseline trajectory shape, S2 shows moderate degradation, and S3 exhibits fundamental segmentation breakdown with the mean trajectory initiating from a non-zero slope.

**Figure A7:**
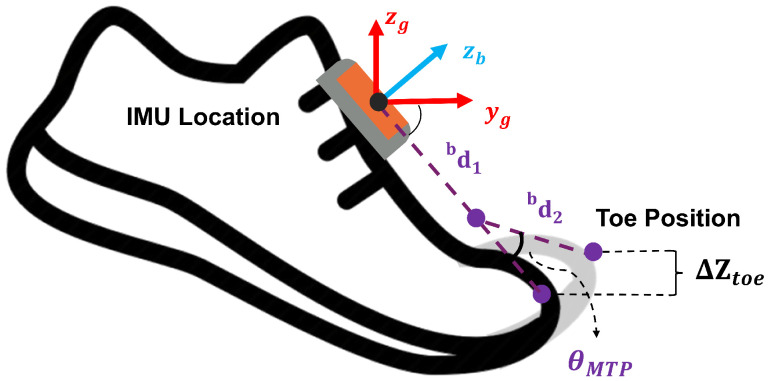
Two-segment foot model for MTP joint flexibility analysis. The total IMU-to-toe distance $L=d_{1}+d_{2}$ is partitioned into a rigid segment d1b from the IMU to the MTP joint, and a phalanx segment d2b from the MTP joint to the toe tip. The MTP flexion angle $\theta_{\mathrm{MTP}}$ makes the rotation of d2b relative to the foot body frame, producing a vertical displacement error $\Delta z_{\mathrm{toe}}$ relative to the rigid-body assumption.

## Data Availability

The data presented in this study are available on request from the corresponding author.

## Appendix A. Derivation of Velocity and Position Bias Equations

This appendix provides the algebraic derivation of the velocity and position difference equations summarized in Section 2.1.1 and Section 2.1.2.

### Appendix A.1. Velocity Correction Derivation

The corrected acceleration, accounting for the acceleration bias $a_{bias}$, is expressed as:

(A1) acorrg(k)=Rbg(k)×(ab(k)−abias)−gg

Using this corrected acceleration, the corrected linear velocity is computed through integration:

(A2) vcorrg(k)=vcorrg(k−1)+Rbg(k)×(ab(k)−abias)−gg×Δt

To establish the kinematic relationship between uncorrected and corrected velocities, the incremental velocity difference δvg(k) is defined as the change in velocity at sample *k* due to the acceleration bias:

(A3) δvg(k)=vg(k)−vcorrg(k)−vg(k−1)−vcorrg(k−1)

Substituting Equations (A1) and (A2) into Equation (A3), the incremental velocity difference simplifies to:

(A4) δvg(k)=Rbg(k)·abias·Δt

The cumulative velocity difference Δvg(k) from the start of the stride to sample *k* is obtained by summing the incremental differences:

(A5) Δvg(k)=∑j=1kδvg(j)=vg(k)−vcorrg(k)=La(k)·abias·Δt,La(k)=∑j=1kRbg(j)

Adding the heel strike impulse term (active for $k>k_{hs}$) to account for the velocity discontinuity at heel contact yields Equation (3) in the main text.

### Appendix A.2. Position Correction Derivation

The corrected position, accounting for acceleration bias, is expressed as:

(A6) pcorrg(k)=pcorrg(k−1)+vcorrg(k−1)·Δt+12Rbg(k)·(ab(k)−abias)−g·Δt2

The incremental position difference δpg(k), representing the change in position at sample *k* attributable to the acceleration bias, is:

(A7) δpg(k)=pg(k)−pcorrg(k)−pg(k−1)−pcorrg(k−1)

Substituting the velocity difference from Equation (A5) into the position integration equation, the incremental position difference can be expressed in terms of the acceleration bias:

(A8) δpg(k)=Δvg(k−1)·Δt+12Rbg(k)·abias·Δt2=La(k)−12Rbg(k)·abias·Δt2

The cumulative position difference up to sample *k* is obtained by summing the incremental differences:

(A9) Δpg(k)=∑k=1kδpg(k)=Lv(k)·abias·Δt2,Lv(k)=∑k=1kLa(k)−12Rbg(k)

Since the heel strike occurs at a specific instant ($k_{hs}$), its effect propagates through all subsequent samples proportionally to the elapsed time since heel strike. Adding this contribution to Equation (A9) yields the complete position difference expression, which is Equation (5) in the main text.

Applying the zero-velocity constraint (Assumption 2) and the ZHC constraint (Assumption 3) to these expressions at k=N then produces the 4×4 matrix system in Equation (8).

## Appendix B. Threshold Optimization

This supervised optimization step uses labeled training data to find the threshold multipliers that maximize classification accuracy. The data were based on the training group participants. The search space is designed such that $k_{1}$ controls ramp detection sensitivity (finer resolution) while $k_{2}$ controls stair detection (coarser resolution, starting where $k_{1}$ ends). The constraint $k_{2}>k_{1}$ ensures proper hierarchical ordering of activity classes.

To determine the optimal threshold multipliers, a grid search is performed over candidate values:

(A10) $$\begin{array}{cc} K_{1} & =\{k:k\in [0,3.5],\;\mathrm{step}\;0.1\}\\ K_{2} & =\{k:k\in [3.5,10],\;\mathrm{step}\;0.5\} \end{array}$$

For each pair $\left(k_{1},k_{2}\right)$ with the constraint $k_{2}>k_{1}$, the classification accuracy is computed against labeled ground truth data:

(A11) $$A\left(k_{1},k_{2}\right)=\frac{1}{N}\sum_{i=1}^{N}1\left[C\left(i\right)=L\left(i\right)\right]$$

where 1[·] is the indicator function that equals 1 when the predicted class C(i) matches the true label L(i), and 0 otherwise.

The optimal threshold pair is selected as:

(A12) $$\left(k_{1}^{*},k_{2}^{*}\right),k_{2}>k_{1},\left(k_{1}^{*},k_{2}^{*}\right)=\arg\max_{\left(k_{1},k_{2}\right)}A\left(k_{1},k_{2}\right)$$

The optimization of threshold multipliers $k_{1}$ and $k_{2}$ was performed through a grid search over the candidate parameter spaces. Figure A1 presents the heat map of classification accuracy across all combinations of $k_{1}$ and $k_{2}$ values evaluated on the training dataset. The optimal parameter combination $\left(k_{1}^{*},k_{2}^{*}\right)=\left(0.89,2.95\right)$ achieved a maximum classification accuracy of 97.4%, as indicated by the asterisk marker. This accuracy demonstrates the effectiveness of the adaptive thresholding approach in distinguishing among the five activities. These optimized threshold multipliers, determined through offline processing of labeled training data, provide robust parameters that can be deployed for real-time activity classification.

For threshold implementation, using the optimized threshold multipliers $k_{1}^{*}$ and $k_{2}^{*}$, the final classification thresholds are computed:

(A13) $$\begin{array}{cc} \mathrm{For}\mathrm{Ascent}: & \left\{\begin{array}{c} T_{ramp}^{+}=\mu_{hs}+k_{1}^{*}\times \sigma_{hs}\\ T_{stair}^{+}=\mu_{hs}+k_{2}^{*}\times \sigma_{hs} \end{array}\right.\\ \mathrm{For}\mathrm{Descent}: & \left\{\begin{array}{c} T_{ramp}^{-}=\mu_{hs}-k_{1}^{*}\times \sigma_{hs}\\ T_{stair}^{-}=\mu_{hs}-k_{2}^{*}\times \sigma_{hs} \end{array}\right. \end{array}$$

These final thresholds incorporate the data-driven optimal multipliers and are applied to classify all strides in the dataset using the classification rule from Equation (15). The thresholds adapt to the specific characteristics of each trial through the baseline $\mu_{hs}$ and spread $\sigma_{hs}$, while maintaining consistent separation criteria defined by $k_{1}^{*}$ and $k_{2}^{*}$.

## Appendix C. Validation of Heel Strike Detection Method

### Appendix C.1. Timing Consistency Analysis

The heel strike instant ($k_{hs}$) is identified as the sample exhibiting the maximum foot pitch angle within each stride window, based on the biomechanical observation that the foot reaches its maximum forward inclination at or near the instant of heel contact with the ground. While this assumption is well-established for level-ground walking, its reliability across all five locomotion activities requires validation, particularly for stair and ramp negotiation, where foot kinematics differ substantially from level walking. To validate this detection method, the foot pitch angle profiles were examined across all activities, and the timing consistency of the detected heel strike instant was quantified across all strides and participants.

To ensure robust detection and avoid spurious local maxima during the early swing phase, the search for the maximum pitch angle is constrained to the second half of the stride window, beyond 50% of the stride duration. This constraint is consistent with the biomechanical expectation that heel strike occurs during the late swing phase, and is supported by the validation results presented below, where all detected $k_{hs}$ values fall between 68.9% and 86.4% of the gait cycle across all five locomotion activities, well within the constrained search window.

Figure A2 shows a representative foot pitch angle profile for one participant across the normalized gait cycle for all five locomotion activities. The filled triangle (▼) indicates the detected heel strike instant $k_{hs}$ corresponding to the maximum pitch angle within each stride. The profile demonstrates that the maximum pitch angle occurs at a biomechanically consistent and expected location within the gait cycle across all activities, corresponding to the late swing phase when the foot approaches the ground for contact.

To quantify the timing consistency of the detected heel strike across all participants and strides, Table A1 presents the peak pitch angle magnitude, the normalized timing of the detected heel strike ($k_{hs}$ as percentage of gait cycle), and the within-stride standard deviation across all 10 participants and 5102 total strides.

**Table A1:** Summary of heel strike detection timing across all five locomotion activities (N = 10 participants, 5102 total strides). Peak timing is expressed as percentage of the normalized gait cycle. Values are reported as mean ± SD.

Activity	N Cycles	Peak Angle (°)	Peak Timing (% GC)	Within-Stride SD (°)
Level Walk	2835	21.92±5.21	68.9±1.4	5.10±0.77
Ramp Ascend	477	19.43±6.27	71.3±1.7	6.20±4.93
Ramp Descend	524	22.08±5.77	68.1±1.8	3.84±0.58
Stair Ascend	634	−0.55±2.93	75.9±2.9	5.95±0.94
Stair Descend	632	0.31±1.38	86.4±3.0	2.69±0.62

The results demonstrate consistent heel strike detection timing across all activities. For level walking, ramp ascent, and ramp descent, the peak pitch angle occurs at 68.9±1.4%, 71.3±2.7%, and 68.1±1.8% of the gait cycle, respectively, with notably low standard deviations confirming highly consistent detection across participants and strides. Stair ascent shows a slightly later peak at 75.9±3.8%, reflecting the modified foot kinematics required to clear the step edge. Stair descent exhibits the latest peak timing at 86.4±3.0%, which is biomechanically expected. During stair descent, the foot is lowered in a controlled manner onto the next step rather than striking it with forward momentum, resulting in a later and lower-magnitude peak pitch angle, as evidenced by the near-zero peak angles (0.31±1.38°) compared with other activities.

The key validation argument lies in the standard deviation of peak timing: if the maximum pitch angle did not reliably correspond to heel strike, the detected $k_{hs}$ timing would vary randomly across strides and participants, producing high standard deviations. The consistently low standard deviations observed across all five activities and 5102 strides confirm that the maximum pitch angle reliably occurs at the same phase of the gait cycle regardless of participant, stride, or locomotion condition, validating the robustness of this detection method across all five activities examined in this study.

### Appendix C.2. Robustness to Heel Strike Timing Uncertainty

While the timing consistency analysis in the previous section demonstrates that the maximum pitch angle method reliably detects heel strike, a residual timing uncertainty of up to ±3.0% GC remains. Since $v_{\mathrm{error},\mathrm{hs}}$ is computed from the kinematic model using $k_{hs}$ as an input, any timing error could potentially affect both the heel strike velocity error. To quantify the impact of this residual uncertainty on the system output, a sensitivity analysis was performed by artificially perturbing $k_{hs}$ by ±1%, ±2%, and ±3% of the gait cycle, selected to span the worst-case timing variability observed in Table A1, where stair descent exhibited the largest standard deviation of ±3.0% GC. For each perturbation level, $v_{\mathrm{error},\mathrm{hs}}$ was recomputed and the results are presented in Figure A3 and Table A2.

**Table A2:** Mean ± SD of $v_{\mathrm{error},\mathrm{hs}}$ (m/s) under heel strike timing perturbations of ±1%, ±2%, and ±3% of the gait cycle across all five locomotion activities. The nominal (unperturbed) values are shown in bold.

Activity	$k_{\mathrm{hs}}-3\%$	$k_{\mathrm{hs}}-2\%$	$k_{\mathrm{hs}}-1\%$	$k_{\mathrm{hs}}^{*}$ (Nominal)	$k_{\mathrm{hs}}+1\%$	$k_{\mathrm{hs}}+2\%$	$k_{\mathrm{hs}}+3\%$
Level Walk	+0.006±0.019	+0.001±0.030	+0.002±0.020	+0.001±0.013	+0.003±0.022	+0.007±0.023	+0.007±0.014
Ramp Ascend	+0.224±0.023	+0.233±0.024	+0.133±0.024	+0.173±0.019	+0.153±0.027	+0.265±0.029	+0.277±0.031
Ramp Descend	−0.253±0.046	−0.364±0.048	−0.264±0.048	−0.193±0.038	−0.390±0.054	−0.304±0.057	−0.322±0.061
Stair Ascend	+0.832±0.305	+0.771±0.326	+0.571±0.126	+0.590±0.109	+0.563±0.178	+0.917±0.111	+1.078±0.250
Stair Descend	−1.022±0.273	−1.083±0.326	−0.983±0.296	−0.740±0.129	−0.733±0.357	−0.829±0.454	−1.447±0.256

To assess the robustness of the kinematic model to potential timing uncertainty in heel strike detection, a sensitivity analysis was performed by artificially perturbing $k_{hs}$ by ±1%, ±2%, and ±3% of the gait cycle. These perturbation levels were selected to reflect the worst-case timing variability observed in Table A1, where stair ascent exhibited the largest standard deviation in peak timing of ±3.8% GC. For each perturbation level, $v_{\mathrm{error},\mathrm{hs}}$ was recomputed and the results are presented in Figure A3 and Table A2.

First, the nominal values ($k_{hs}^{*}$) in Figure A3 are consistent with the baseline results reported in Figure 8, confirming that the sensitivity analysis is performed on the same data and that the perturbation framework is correctly implemented. The results demonstrate that the system is robust to realistic heel strike timing uncertainty across all five locomotion activities. For level walking, changes in $v_{\mathrm{error},\mathrm{hs}}$ remained negligible across all perturbation levels (maximum change of 0.006 m/s), well below the level-ramp classification threshold gap of approximately 0.172 m/s. For ramp activities, although larger absolute changes were observed (up to 0.197 m/s for ramp descent at +1%), the sign and order of magnitude of $v_{\mathrm{error},\mathrm{hs}}$ were preserved across all perturbation levels, maintaining correct directional classification. For stair activities, while the largest absolute changes were observed due to the higher baseline magnitude, the sign of $v_{\mathrm{error},\mathrm{hs}}$ was consistently preserved across all perturbation levels, confirming that ascending and descending stair activities would be correctly distinguished regardless of timing uncertainty. Most critically, no perturbation caused $v_{\mathrm{error},\mathrm{hs}}$ to cross a classification boundary into an incorrect activity region, confirming that the maximum pitch angle heel strike detection method produces classification-stable outputs under realistic timing uncertainty of up to ±3% of the gait cycle corresponding to the worst-case variability observed across all activities and participants.

## Appendix D. Effect of Calibration Quality on Classification Performance

To evaluate the robustness of the activity classification framework to inaccurate or unavailable calibration, a sensitivity analysis was conducted across four calibration scenarios of progressively degrading quality with just one participant in the same environment for a real-time experiment. In the nominal scenario (S1), the baseline parameter $\mu_{hs}$ was computed from 20 strides of pure level-ground walking as described in Section 2.2.2, yielding well-separated ${\hat{v}}_{error,hs}$ distributions across all five activity types and an overall classification accuracy of 96.8%. In the second scenario (S2), the calibration window was contaminated by replacing half of the level-ground strides with ramp ascent strides, simulating a user who begins walking on a mild incline during the calibration phase. This shifted $\mu_{hs}$ positively, compressing the ${\hat{v}}_{error,hs}$ for ramp ascent toward zero and pushing level walk into negative territory, reducing the separation between these two activities. Classification accuracy decreased to 82.1%, with the primary confusion occurring between level walk and ramp ascent. In the third scenario (S3), the calibration window was contaminated with stair ascent strides, producing a larger positive shift in $\mu_{hs}$ and a wider $\sigma_{hs}$. This caused ramp ascent ${\hat{v}}_{error,hs}$ values to cross zero and fall into the level-ground classification region, while stair ascent values shifted toward the level-ground boundary. Overall accuracy dropped to 68.2%, with misclassifications spread across multiple activity pairs. Figure A4 illustrates the progressive shift in ${\hat{v}}_{error,hs}$ distributions across the four calibration scenarios, demonstrating how increasing calibration contamination compresses inter-activity separation and displaces classification boundaries.

In the fourth scenario (S4), no individual calibration was performed and $\mu_{hs}$ was set to the mean of all five activity types combined, representing the worst-case condition of a completely uninformative baseline. The ${\hat{v}}_{error,hs}$ values for level walk and ramp ascent were displaced into positive territory, inverting the expected sign pattern and collapsing the separation between ascending and level activities. Classification accuracy fell to 46.1%, approaching chance-level performance for some activity pairs. Figure A5 presents the confusion matrices for all four scenarios, confirming the progressive degradation in classification performance as calibration quality decreases.

These results demonstrate that the classification framework degrades gracefully with increasing calibration contamination. However, complete absence of level-ground calibration substantially degrades performance, confirming that the initial calibration phase on level ground is an important practical requirement for deployment. In real-world scenarios where users begin on level ground before transitioning to non-level terrain, the calibration procedure is readily achievable and represents a practical constraint consistent with standard wearable system initialization protocols.

## Appendix E. Sensitivity Analysis of ZVP Detection Parameters

To evaluate the robustness of the ZVP detection algorithm to its four tunable parameters, a sensitivity analysis was performed by perturbing each parameter by 100% from its empirically determined baseline value ($T_{a}=15\;{m/s}^{2}$, $T_{\omega }=4\;\mathrm{rad}/s$, $W_{cap}=8\;\mathrm{samples}$, $T_{max}=80\;\mathrm{ms}$). The algorithm relies on four parameters with distinct roles: $T_{a}$ and $T_{\omega }$ serve as qualification gates admitting samples only when both acceleration and angular velocity fall below their respective thresholds; $W_{cap}$ defines the minimum candidate count before ZVP selection; and $T_{max}$ sets the maximum accumulation window duration before a forced detection. Three perturbation scenarios were defined: in S1, qualification thresholds were increased by 100% ($T_{a}=30\;{m/s}^{2}$, $T_{\omega }=8\;\mathrm{rad}/s$); in S2, window capacity was halved ($W_{cap}=4\;\mathrm{samples}$); and in S3, maximum window duration was halved ($T_{max}=40\;\mathrm{ms}$). The protocol involved three participants walking approximately 4 min on level ground at normal, faster, and slower speeds and in different footwear to ensure gait variability. The results are shown in Figure A6.

The results demonstrate distinct degradation patterns consistent with the algorithmic role of each parameter, with heel strike timing assessed using the maximum pitch angle criterion described in Appendix C. In S1, doubling $T_{a}$ and $T_{\omega }$ enlarged the candidate pool but left the selected minimum angular velocity sample unchanged, resulting in only modest variability increase from 1.5% to 3.8% with stable mean HS location at 67.6% and well-preserved trajectory shape, confirming robustness of the argmin selection mechanism. In S2, halving $W_{cap}$ caused premature window closure, committing to a ZVP before the foot reached its quietest moment and increasing variability to 5.9% with a small mean shift to 66.6%. In S3, halving $T_{max}$ to 35 ms, approximately 2 samples at 60 Hz, produced a fundamental breakdown: the mean trajectory initiates from a non-zero slope rather than a stationary reference, directly contradicting the zero-velocity definition and confirming that detections occur outside the true flat-foot phase. This yielded the most severe degradation, with variability reaching 11.2% and mean HS shifting to 66.4%. These results validate the empirically chosen baseline values, as perturbations to $T_{a}$ and $T_{\omega }$ produce only gradual degradation, while aggressive reduction in $T_{max}$ leads to incorrect stride segmentation.

## Appendix F. Numerical Stability of the Constraint Matrix

The solution of the bias parameters $a_{bias}$ and $v_{error,hs}$ requires inverting the 4×4 matrix M in Equation (8). The matrix M is constructed from two types of quantities: (1) *N* and $k_{hs}$, which are fixed natural numbers for each stride determined by gait segmentation, and (2) the cumulative rotation matrix sums $L_{a}\left(N\right)$ and $L_{v,(3,:)}\left(N\right)$, which are derived from IMU-measured orientation angles and are therefore subject to sensor noise. To assess whether realistic IMU noise could destabilize the inversion, the condition number κ(M) was evaluated across 677 strides, including all the activities under four scenarios: S0 (nominal, no added noise), S1 (σ=0.01 m/s^2^), S2 (σ=0.05 m/s^2^), and S3 (σ=0.10 m/s^2^), where small random orientation perturbations were applied (equivalent to additive noise in roll/pitch/yaw), and the corresponding perturbed rotation matrices were recomputed.

As shown in Table A3, the median condition number was κ=8.4 across all scenarios, with a maximum of κ=520.3 in the nominal case, and no stride exceeded a conservative ill-conditioning threshold of κ>1000 under any noise level. Critically, the condition number was insensitive to added noise across all scenarios, with negligible variation in both mean and maximum κ. This robustness follows from the biomechanical structure of gait: $L_{a}$ and $L_{v,(3,:)}$ are cumulative sums of rotation matrices over a full stride of 60–80 samples, providing natural noise averaging that prevents individual sample perturbations from destabilizing the accumulated Jacobian. Together, these results confirm that M is numerically well-conditioned under realistic sensor noise conditions, and that the matrix inversion in Equation (8) is stable throughout all locomotion activities examined in this study.

**Table A3:** Numerical stability of the 4×4 ZHC constraint matrix M (Equation (8)) across sensor noise scenarios. κ(M) = condition number evaluated over 677 strides; ill-conditioned threshold κ>1000.

Scenario	$\sigma_{\mathrm{noise}}$ (m/s^2^)	κ Mean ± SD	κ Median	κ Max
S0 (nominal)	0	9.3±19.7	8.4	520.3
S1	0.01	9.3±19.7	8.4	519.9
S2	0.05	9.3±19.7	8.4	520.0
S3	0.10	9.3±19.5	8.4	516.8

## Appendix G. Assumption for Toe Position Estimation

The toe position estimation in Section 2.5 assumes the foot behaves as a rigid body, treating the IMU-to-toe displacement vector db=[0d0]T as fixed throughout the gait cycle. While reasonable during mid-swing, this simplification does not capture metatarsophalangeal (MTP) joint flexion, which occurs primarily at toe-off. To quantify the impact of this assumption, the rigid-body model was extended to a two-segment formulation, as illustrated in Figure A7. The total IMU-to-toe distance $d=d_{1}+d_{2}$ cm is partitioned into a rigid segment d1b=[0d10]T from the IMU to the MTP joint, and a phalanx segment d2b=[0d20]T from the MTP joint to the toe tip. Under this formulation, the flexible toe position is:

(A14) ptoeg=pIMUg+Rbg·d1b+Rbg·RMTP(θ)·d2b

where $R_{\mathrm{MTP}}\left(\theta \right)$ is the sagittal-plane rotation matrix about the MTP joint axis. The vertical height error introduced by the rigid-body assumption is:

(A15) Δztoe=Rbg·RMTP(θ)−I·d2bz

To bound this error across realistic conditions, $\Delta z_{\mathrm{toe}}$ was evaluated using stride data from one representative participant with a measured IMU-to-toe distance of $L=d_{1}+d_{2}=15$ cm, covering all five locomotion activities. Three phalanx lengths ($d_{2}=1,2,3$ cm) and three MTP flexion angles spanning the reported in-vivo range of $45^{\circ }$–$70^{\circ }$ required during normal gait [86,87] were evaluated: $\theta =15^{\circ }$ (conservative mid-swing estimate), $\theta =45^{\circ }$ (lower bound of functional requirement), and $\theta =60^{\circ }$ (mid-range of reported dorsiflexion). The rotation matrix Rbg was taken from IMU orientation estimates at the mid-swing instant of each stride, as mid-swing is the phase at which minimum foot clearance is assessed.

Table A4 reports $\Delta z_{\mathrm{toe}}$ across all conditions, representing the expected toe height error if the rigid-body assumption were relaxed and MTP joint flexibility were accounted for. At $\theta =15^{\circ }$, errors ranged from 0.03±0.03 cm to 0.39±0.01 cm across all activities and $d_{2}$ values. Even at $\theta =60^{\circ }$, the largest error reached 1.44±0.06 cm, which, while approaching the toe stride height variability of ±1.2 cm reported in Section 4.5, represents a worst-case upper bound corresponding to maximum MTP flexion. At the clinically relevant mid-swing instant ($\theta =15^{\circ }$), errors remained below 0.39 cm across all activities and phalanx lengths, well within system uncertainty. These results suggest that the rigid-body assumption introduces limited additional error in toe clearance estimation at the mid-swing instant relevant for trip risk assessment.

**Table A4:** Effect of MTP joint flexibility on toe height estimation error ($\Delta z_{\mathrm{toe}}$, cm, mean ± SD) across locomotion activities, MTP flexion angles, and phalanx segment lengths ($d_{2}$).

	$\theta_{\mathrm{MTP}}=15^{\circ }$			$\theta_{\mathrm{MTP}}=45^{\circ }$			$\theta_{\mathrm{MTP}}=60^{\circ }$		
**Act.**	$d_{2}=1$ **cm**	$d_{2}=2$ **cm**	$d_{2}=3$ **cm**	$d_{2}=1$ **cm**	$d_{2}=2$ **cm**	$d_{2}=3$ **cm**	$d_{2}=1$ **cm**	$d_{2}=2$ **cm**	$d_{2}=3$ **cm**
LW	0.11±0.09	0.21±0.17	0.32±0.26	0.31±0.27	0.62±0.54	0.92±0.81	0.41±0.29	0.81±0.58	1.22±0.87
RA	0.13±0.00	0.26±0.00	0.39±0.01	0.38±0.01	0.76±0.02	1.13±0.03	0.48±0.02	0.96±0.04	1.44±0.06
RD	0.03±0.03	0.06±0.06	0.10±0.08	0.02±0.09	0.05±0.19	0.07±0.28	0.08±0.12	0.17±0.23	0.25±0.35
SA	0.04±0.05	0.08±0.09	0.12±0.14	0.19±0.15	0.38±0.31	0.57±0.46	0.30±0.20	0.60±0.39	0.89±0.59
SD	0.04±0.03	0.09±0.06	0.13±0.09	0.22±0.09	0.43±0.18	0.65±0.28	0.33±0.11	0.67±0.22	1.00±0.33

## References

[1] Czech M.D., Psaltos D., Zhang H., Adamusiak T., Calicchio M., Kelekar A., Messere A., Dijk K.V., Ramos V., Demanuele C. (2020). Age and environment-related differences in gait in healthy adults using wearables. npj Digit. Med..

[2] Seifallahi M., Lahiri S., Galvin J.E., Ghoraani B. (2026). Technology-Enhanced Dual-Task Testing for Alzheimer’s Disease and Related Dementias: A Review of Trends, Tools, and Emerging Directions. IEEE Trans. Neural Syst. Rehabil. Eng..

[3] Joshi M., Ashrafian H., Aufegger L., Khan S., Arora S., Cooke G., Darzi A. (2019). Wearable sensors to improve detection of patient deterioration. Expert Rev. Med. Devices.

[4] Nassajpour M., Seifallahi M., Rosenfeld A., Tolea M.I., Galvin J.E., Ghoraani B. (2025). Comparison of wearable and depth-sensing technologies with electronic walkway for comprehensive gait analysis. Sensors.

[5] Ghoreishi N., LaCourse J., Arthanat S., LaRoche D., Chen D. (2025). Auto-Calibrated Wearable System for Load Vertical Location Estimation During Manual Lifting. IEEE Internet Things J..

[6] Vo D.K., Trinh K. (2024). Advances in Wearable Biosensors for Healthcare: Current Trends, Applications, and Future Perspectives. Biosensors.

[7] Almujally N., Khan D., Mudawi N.A., Alonazi M., Alhasson H.F., Jalal A., Liu H. (2025). Wearable sensors-based assistive technologies for patient health monitoring. Front. Bioeng. Biotechnol..

[8] Nassajpour M., Shuqair M., Rosenfeld A., Tolea M.I., Galvin J.E., Ghoraani B. (2024). Objective estimation of m-CTSIB balance test scores using wearable sensors and machine learning. Front. Digit. Health.

[9] Seifallahi M., Galvin J.E., Ghoraani B. (2024). Detection of mild cognitive impairment using various types of gait tests and machine learning. Front. Neurol..

[10] Hosseinalizadeh M., Asghari M., Toosizadeh N. (2024). Sensor-Based Frailty Assessment Using Fitbit. Sensors.

[11] Haagsma J.A., Olij B.F., Majdan M., Van Beeck E.F., Vos T., Castle C.D., Dingels Z.V., Fox J.T., Hamilton E.B., Liu Z. (2020). Falls in older aged adults in 22 European countries: Incidence, mortality and burden of disease from 1990 to 2017. Inj. Prev..

[12] Adam C.E., Fitzpatrick A.L., Leary C.S., Ilango S.D., Phelan E.A., Semmens E.O. (2024). The impact of falls on activities of daily living in older adults: A retrospective cohort analysis. PLoS ONE.

[13] Usmani S., Saboor A., Haris M., Khan M.A., Park H. (2021). Latest Research Trends in Fall Detection and Prevention Using Machine Learning: A Systematic Review. Sensors.

[14] Buzpınar M.A. (2025). Fall Detection and Prevention Systems: Sensor Type Perspective. Osman. Korkut Ata Üniv. Fen Bilim. Enst. Derg..

[15] Montero-Odasso M., Van Der Velde N., Martin F.C., Petrovic M., Tan M.P., Ryg J., Aguilar-Navarro S., Alexander N.B., Becker C., Blain H. (2022). World guidelines for falls prevention and management for older adults: A global initiative. Age Ageing.

[16] Khiyara I., Sidaway B., Hejrati B. (2025). Utilizing Rhythmic Haptic Cueing in Arm Swing Training to Improve Gait Speed Among Older Adults. Ann. Biomed. Eng..

[17] Noghani M.A., Sharafian E., Sidaway B., Hejrati B. (2025). Increasing thigh extension with haptic feedback affects leg coordination in young and older adult walkers. J. Biomech..

[18] Sharafian M.E., Ellis C., Sidaway B., Hayes M., Hejrati B. (2025). The effects of real-time haptic feedback on gait and cognitive load in older adults. IEEE Trans. Neural Syst. Rehabil. Eng..

[19] Khiyara I., Sidaway B., Hejrati B. (2026). A Wearable Haptic Feedback System for Arm-Swing Amplitude Modulation During Overground Walking in Older Adults. Sensors.

[20] Khiyara I., Sidaway B., Hejrati B. (2025). Modulating arm swing via haptic cueing alters interlimb neural coupling in older adults. Front. Physiol..

[21] Nagano H. (2022). Gait Biomechanics for Fall Prevention among Older Adults. Appl. Sci..

[22] Begg R., Best R., Dell’Oro L., Taylor S. (2007). Minimum foot clearance during walking: Strategies for the minimisation of trip-related falls. Gait Posture.

[23] Al Bochi A., Delfi G., Dutta T. (2021). A Scoping Review on Minimum Foot Clearance: An Exploration of Level-Ground Clearance in Individuals with Abnormal Gait. Int. J. Environ. Res. Public Health.

[24] Labott B.K., Herold F., Langhans C., Halfpaap N., Grässler B., Hökelmann A., Müller N.G., Hamacher D. (2025). Minimum toe clearance variability in older adults with mild cognitive impairment: Differences to healthy controls and effects of a dance intervention. Gait Posture.

[25] Gelinne A.M., Shelton A.D., Franz J.R. (2025). The impact of slip perturbations on minimum toe clearance during walking in younger and older adults. PLoS ONE.

[26] Fehr K.H., Bartloff J.N., Wang Y., Hetzel S., Adamczyk P.G. (2024). Estimation of minimum foot clearance using a single foot-mounted inertial sensor and personalized foot geometry scan. Sci. Rep..

[27] Fan B., Li Q., Liu T. (2020). Accurate foot clearance estimation during level and uneven ground walking using inertial sensors. Meas. Sci. Technol..

[28] Prisco G., Pirozzi M.A., Santone A., Esposito F., Cesarelli M., Amato F., Donisi L. (2024). Validity of Wearable Inertial Sensors for Gait Analysis: A Systematic Review. Diagnostics.

[29] Prasanth H., Caban M., Keller U., Courtine G., Ijspeert A., Vallery H., von Zitzewitz J. (2021). Wearable Sensor-Based Real-Time Gait Detection: A Systematic Review. Sensors.

[30] Mobbs R.J., Perring J., Raj S.M., Maharaj M., Yoong N.K.M., Sy L.W., Fonseka R.D., Natarajan P., Choy W.J. (2022). Gait metrics analysis utilizing single-point inertial measurement units: A systematic review. mHealth.

[31] Luo Y., Coppola S.M., Dixon P.C., Li S., Dennerlein J.T., Hu B. (2020). A database of human gait performance on irregular and uneven surfaces collected by wearable sensors. Sci. Data.

[32] Yuan S., Zhang Y., Shi Y., Li Z. (2024). A Novel ESKF-Based ZUPT Using Midpoint Integration Approach for Indoor Pedestrian Navigation. IEEE Sens. J..

[33] Wang Y., Fehr K.H., Adamczyk P.G. (2024). Impact-Aware Foot Motion Reconstruction and Ramp/Stair Detection Using One Foot-Mounted Inertial Measurement Unit. Sensors.

[34] Zhang W., Wei D., Yuan H. (2020). The Improved Constraint Methods for Foot-Mounted PDR System. IEEE Access.

[35] Ju H., Lee M.S., Park S.Y., Song J.W., Park C.G. (2015). A pedestrian dead-reckoning system that considers the heel-strike and toe-off phases when using a foot-mounted IMU. Meas. Sci. Technol..

[36] Wagner J.F., Kohl M., Gyorfi B. (2022). Reevaluation of Algorithmic Basics for ZUPT-Based Pedestrian Navigation. IEEE Access.

[37] Tong X., Su Y., Li Z., Si C., Han G., Ning J., Yang F. (2020). A Double-Step Unscented Kalman Filter and HMM-Based Zero-Velocity Update for Pedestrian Dead Reckoning Using MEMS Sensors. IEEE Trans. Ind. Electron..

[38] Huang Z., Ye G., Yang P., Yu W. (2025). Application of multi-sensor fusion localization algorithm based on recurrent neural networks. Sci. Rep..

[39] Liu W., Caruso D., Ilg E., Dong J., Mourikis A.I., Daniilidis K., Kumar V., Engel J. (2020). TLIO: Tight Learned Inertial Odometry. IEEE Robot. Autom. Lett..

[40] Li J., Zhou X., Qiu S., Mao Y., Wang Z., Loo C.K., Liu X. (2024). Learning-Based Stance Phase Detection and Multisensor Data Fusion for ZUPT-Aided Pedestrian Dead Reckoning System. IEEE Internet Things J..

[41] Nisticò Y., Kim H., Soares J.C.V., Fink G., Park H.W., Semini C. (2025). Multi-Sensor Fusion for Quadruped Robot State Estimation Using Invariant Filtering and Smoothing. IEEE Robot. Autom. Lett..

[42] Karle P., Fent F., Huch S., Sauerbeck F., Lienkamp M. (2023). Multi-Modal Sensor Fusion and Object Tracking for Autonomous Racing. IEEE Trans. Intell. Veh..

[43] Xu R., Chen S., Bai S., Wen W. (2024). Nonlinearity-Aware ZUPT-Aided Pedestrian Inertial Navigation Based on Cubature Kalman Filter in Urban Canyons. IEEE Trans. Instrum. Meas..

[44] Bucci A., Franchi M., Ridolfi A., Secciani N., Allotta B. (2023). Evaluation of UKF-Based Fusion Strategies for Autonomous Underwater Vehicles Multisensor Navigation. IEEE J. Ocean. Eng..

[45] Subramaniam S., Faisal A.I., Deen M.J. (2022). Wearable Sensor Systems for Fall Risk Assessment: A Review. Front. Digit. Health.

[46] Chen S., Zhu C., Chen X., Yi J. (2025). Machine Learning-Based Real-Time Walking Activity and Posture Estimation in Construction With a Single Wearable Inertial Measurement Unit. IEEE Trans. Autom. Sci. Eng..

[47] Luo S., Shu X., Zhu H., Yu H. (2023). Early Prediction of Lower Limb Prostheses Locomotion Mode Transition Based on Terrain Recognition. IEEE Sens. J..

[48] Sharafian M.E., Ellis C., Hejrati B. (2025). Real-Time Activity Recognition Using Minimal Biomechanical Features: A Lightweight IMU-Based Classifier for Older Adults. IEEE Access.

[49] Wilson T., Wisdish S., Osofa J., Farris D.J. (2025). Evaluating Machine Learning-Based Classification of Human Locomotor Activities for Exoskeleton Control Using Inertial Measurement Unit and Pressure Insole Data. Sensors.

[50] Zhang S., Li Y., Zhang S., Shahabi F., Xia S., Deng Y., Alshurafa N. (2022). Deep Learning in Human Activity Recognition with Wearable Sensors: A Review on Advances. Sensors.

[51] Hutabarat Y., Owaki D., Hayashibe M. (2021). Recent Advances in Quantitative Gait Analysis Using Wearable Sensors: A Review. IEEE Sens. J..

[52] Lu Y., Zhu J., Chen W., Ma X. (2023). An IMU-Based Real-Time Gait Detection Method for Intelligent Control of Knee Assistive Devices. IEEE Trans. Instrum. Meas..

[53] Al-qaness M.A., Dahou A., Abd Elaziz M., Helmi A.M. (2024). Human activity recognition and fall detection using convolutional neural network and transformer-based architecture. Biomed. Signal Process. Control..

[54] Ngu A.H., Metsis V., Coyne S., Srinivas P., Salad T., Mahmud U., Chee K.H. (2022). Personalized Watch-Based Fall Detection Using a Collaborative Edge-Cloud Framework. Int. J. Neural Syst..

[55] Choi A., Kim T.H., Yuhai O., Jeong S., Kim K., Kim H., Mun J.H. (2022). Deep Learning-Based Near-Fall Detection Algorithm for Fall Risk Monitoring System Using a Single Inertial Measurement Unit. IEEE Trans. Neural Syst. Rehabil. Eng..

[56] González-Cañete F.J., Casilari E. (2020). Consumption Analysis of Smartphone based Fall Detection Systems with Multiple External Wireless Sensors. Sensors.

[57] Zafar R.O., Zafar F. (2025). Real-time activity and fall detection using transformer-based deep learning models for elderly care applications. BMJ Health Care Inform..

[58] Forster C., Carlone L., Dellaert F., Scaramuzza D. IMU preintegration on manifold for efficient visual-inertial maximum-a-posteriori estimation. Proceedings of the Robotics: Science and Systems XI.

[59] Sharafian E., Taghvaeipour A., Ghassabzadeh M. (2022). Revisiting screw theory-based approaches in the constraint wrench analysis of robotic systems. Robotica.

[60] Faeghinejad A., Hawthorne L., Hejrati B. (2026). Design and Feasibility Assessment of a Prototype Wearable Upper-Limb Device for Facilitating Arm Swing Training. Actuators.

[61] Moghaddam E.S., Saryazdi M.G., Taghvaeipour A. (2023). Trajectory Optimization of a Spot-Welding Robot in Thejoint and Cartesian Spaces. Int. J. Robot. Autom..

[62] Alcala E., Voerman J., Konrath J., Vydhyanathan A. (2021). Xsens DOT wearable sensor platform white paper. White Pap..

[63] Dadashi F., Mariani B., Rochat S., Büla C.J., Santos-Eggimann B., Aminian K. (2013). Gait and foot clearance parameters obtained using shoe-worn inertial sensors in a large-population sample of older adults. Sensors.

[64] Shaikh U.Q., Shahzaib M., Shakil S., Bhatti F.A., Aamir Saeed M. (2023). Robust and adaptive terrain classification and gait event detection system. Heliyon.

[65] Li H., Derrode S., Pieczynski W. (2019). An adaptive and on-line IMU-based locomotion activity classification method using a triplet Markov model. Neurocomputing.

[66] Tukey J.W. (1977). Exploratory Data Analysis.

[67] Leys C., Ley C., Klein O., Bernard P., Licata L. (2013). Detecting outliers: Do not use standard deviation around the mean, use absolute deviation around the median. J. Exp. Soc. Psychol..

[68] Skog I., Handel P., Nilsson J.O., Rantakokko J. (2010). Zero-velocity detection—An algorithm evaluation. IEEE Trans. Biomed. Eng..

[69] Wagstaff B., Peretroukhin V., Kelly J. (2019). Robust data-driven zero-velocity detection for foot-mounted inertial navigation. IEEE Sens. J..

[70] Wagstaff B., Kelly J. (2018). LSTM-based zero-velocity detection for robust inertial navigation. Proceedings of the 2018 International Conference on Indoor Positioning and Indoor Navigation (IPIN), Nantes, France, 24–27 September 2018.

[71] Barth J., Oberndorfer C., Pasluosta C., Schülein S., Gassner H., Reinfelder S., Kugler P., Schuldhaus D., Winkler J., Klucken J. (2015). Stride segmentation during free walk movements using multi-dimensional subsequence dynamic time warping on inertial sensor data. Sensors.

[72] Chen D., Ghoreishi N., Olugbon F., Ansah S., Huang M.C., Yu Q. (2022). Optimal pressure sensor locations in smart insoles for heel-strike and toe-off detection. Proceedings of the 2022 IEEE Biomedical Circuits and Systems Conference (BioCAS), Taipei, Taiwan, 13–15 October 2022.

[73] Noghani M.A., Shahinpoor M., Hejrati B. (2021). Design and validation of a smartphone-based haptic feedback system for gait training. IEEE Robot. Autom. Lett..

[74] Hossain M.T., Noghani M.A., Sidaway B., Hejrati B. (2023). Investigating the efficacy of a tactile feedback system to increase the gait speed of older adults. Hum. Mov. Sci..

[75] Nagano H., Sparrow W.A., Mizukami K., Sarashina E., Begg R. (2020). Ageing Effects on Tripping Risk: The Foot-Ground Clearance of Healthy Community Dwelling Japanese Cohorts Aged 50, 60 and 70 Years. Res. Sq..

[76] Cohen J. (2013). Statistical Power Analysis for the Behavioral Sciences.

[77] Benoussaad M., Sijobert B., Mombaur K., Azevedo Coste C. (2015). Robust foot clearance estimation based on the integration of foot-mounted IMU acceleration data. Sensors.

[78] Jocham A.J., Laidig D., Guggenberger B., Seel T. (2024). Measuring highly accurate foot position and angle trajectories with foot-mounted IMUs in clinical practice. Gait Posture.

[79] Ullauri J.B., Akiyama Y., Okamoto S., Yamada Y. (2019). Technique to reduce the minimum toe clearance of young adults during walking to simulate the risk of tripping of the elderly. PLoS ONE.

[80] Gao F., Liu G., Liang F., Liao W.H. (2020). IMU-based locomotion mode identification for transtibial prostheses, orthoses, and exoskeletons. IEEE Trans. Neural Syst. Rehabil. Eng..

[81] Kang I., Molinaro D.D., Choi G., Camargo J., Young A.J. (2022). Subject-independent continuous locomotion mode classification for robotic hip exoskeleton applications. IEEE Trans. Biomed. Eng..

[82] Cheng S., Bolívar-Nieto E., Gregg R.D. (2021). Real-time activity recognition with instantaneous characteristic features of thigh kinematics. IEEE Trans. Neural Syst. Rehabil. Eng..

[83] Moura Coelho R., Gouveia J., Botto M.A., Krebs H.I., Martins J. (2022). Real-time walking gait terrain classification from foot-mounted Inertial Measurement Unit using Convolutional Long Short-Term Memory neural network. Expert Syst. Appl..

[84] Zhou H., Zhang X., Feng Y., Zhang T., Xiong L. (2025). Efficient human activity recognition on edge devices using DeepConv LSTM architectures. Sci. Rep..

[85] Bhakta K., Maldonado-Contreras J., Camargo J., Zhou S., Compton W., Herrin K.R., Young A.J. (2025). Continuous-context, user-independent, real-time intent recognition for powered lower-limb prostheses. J. Biomech. Eng..

[86] Nawoczenski D.A., Baumhauer J.F., Umberger B.R. (1999). Relationship between clinical measurements and motion of the first metatarsophalangeal joint during gait. J. Bone Jt. Surg..

[87] Hetherington V.J., Johnson R., Albritton J. (1990). Necessary dorsiflexion of the first metatarsophalangeal joint during gait. J. Foot Surg..

